# Efficacy, acceptability and feasibility of daily text-messaging in promoting glycaemic control and other clinical outcomes in a low-resource setting of South Africa: A randomised controlled trial

**DOI:** 10.1371/journal.pone.0224791

**Published:** 2019-11-27

**Authors:** Eyitayo Omolara Owolabi, Daniel Ter Goon, Anthony Idowu Ajayi

**Affiliations:** 1 Department of Nursing Science, Faculty of Health Sciences, University of Fort Hare, East London, South Africa; 2 Population Dynamics and Reproductive Health Unit, African Population and Health Research Centre, APHRC Campus, Nairobi, Kenya; Weill Cornell Medical College in Qatar, QATAR

## Abstract

South Africa is confronted with a high burden of diabetes, the majority of which are poorly controlled. The use of mHealth, specifically text messaging for fostering health, is evolving and studies on its efficacy, the majority of which were conducted in developed countries, have documented mixed findings. There is no such study done amongst patients living with diabetes in the resource-poor settings of South Africa. The aim of this study is to determine the efficacy, acceptability and feasibility of text-messaging in improving glycaemic control and other clinical outcomes among individuals living with diabetes in low-resource settings in Eastern Cape, South Africa. The study adopted a multi-centre, two-arm, parallel, randomised-controlled trial design. The study was conducted amongst patients with an uncontrolled glycaemic status. Participants were randomly assigned to the intervention (n = 108) and the control arm (n = 108). Participants in the intervention arm received daily educational text messages on diabetes for six months. Data was collected at baseline and six months post-intervention. Blood glucose, blood pressure and anthropometric measurements followed standard procedure. Mixed-model analysis was used to assess the impact of the text messages on blood glucose while linear regression was used to assess its effect on other clinical outcomes such as weight, body mass index, systolic and diastolic blood pressure. The mean age of the participants was 60.64 (SD± 11.58) years. The majority of the participants had a secondary level of education (95.3%) and earned 104.80 to 991.42 USD per month (67.7%). Both arms of the study showed improvement in their blood glucose levels, but the intervention did not have any significant effect, the mean adjusted change in blood glucose was 0.26 (-0.81 to 1.32), p = 0.634. Also, the intervention did not have any significant effect on weight, body mass index, systolic and diastolic blood pressure. Almost all participants (90.74%) were pleased with the intervention and felt it was helpful. Of those who participated in the intervention, 91% completed the follow-up after 6 months. Unidirectional text-messaging was acceptable and feasible amongst adults living with diabetes in this setting. However, its efficacy in improving glycaemic status and other clinical outcomes remains doubtful.

**Trial Registration:** Pan African Clinical Trial Registry PACTR201810599931422.

## Introduction

Diabetes is a significant public health and socio-economic challenge worldwide with an increasing prevalence and burden [1]. It is also a leading cause of disability and death in both the developing and developed countries [2,3]. The burden of diabetes is higher in developing countries where there is a lack of effective and adequately equipped health care systems as well as poor prevention strategies [4]. South Africa ranks second, just after Ethiopia, amongst the African countries with the highest prevalence of diabetes [2]. The burden of diabetes in South Africa as with other nations is further complicated by a high level of sub-optimal control [5]. Several studies conducted in South Africa have recorded a high level of uncontrolled diabetes, ranging from 69.3% in the Northwest Province to as high as 84% in the Eastern Cape Province [6,7].

Various factors have been shown to be associated with a high level of uncontrolled diabetes. These include patients-related factors, like the duration of disease, a poor level of knowledge, a low level of adherence to drugs and the recommended lifestyle regimen, poor self-management behaviour and low self-efficacy [6,8–10]. Other patient-related factors are socio-demographic characteristics, like younger age, a low level of education and income [11]. Health system-related factors such as shortage of manpower, a deficiency in disease management guidelines and poor infrastructures sometimes also impact on the treatment outcomes, including the glycaemic control of patients [10, 12]. Several other cardiovascular risk factors like obesity, physical inactivity, alcohol use and smoking also contribute to the growing burden of the sub-optimal control of diabetes [13,14]. Given the central role of the patients in the management of diabetes, interventions that are aimed at educating and empowering the patients are crucial [15,16]. Traditional measures of educating and empowering the patients through face-to-face interactions are becoming challenging due to the excessive healthcare system workload exacerbated by a shortage of manpower. This is currently being augmented with several forms of distance education using various technological measures [17,18].

The unprecedented rise in mobile technology and the continuous improvement in mobile cellular network coverage across the world have prompted the use of information and communication technology (ICT) measures to advance healthcare[19]. Information and communication technology measures are now being used to educate patients, create awareness, send reminders, share results, collect data, amongst many other functions [19]. Plausibly, improvement in knowledge, which could result from distance education via ICT measures could also bring about an improvement in self-management and self-efficacy of individuals and ultimately promote health [17]. Specifically, the use of text messaging for health promotion is encouraged because of the direct linkage to the recipient, the confidentiality and convenience of the message, as well as the spontaneity of delivery, and the appealing nature [17, 20, 21]. Many of the studies utilising text messaging for promoting health amongst individuals living with diabetes were conducted in developed countries [22–27], a few in developing countries, including some African countries resulting in varied and mixed findings [28–31], but none in South Africa. In addition, the use of text messaging among patients living with diabetes has been sparsely investigated in rural areas. This study, therefore, sought to determine the efficacy, acceptability and feasibility of text messaging in promoting glycaemic control and improvement in other clinical measures amongst patients living with diabetes attending primary health care facilities in resource-poor areas of Eastern Cape, South Africa.

## Materials and methods

The study adopted a multicentre, two-arm, parallel, randomised controlled trial design. The study assessed the effectiveness of a six-month mobile phone SMS intervention in addition to diabetes standard care in promoting glycaemic control and improvement in other clinical outcomes.

### Study setting

The study was conducted at the outpatient departments of six selected primary healthcare centres in two districts in Eastern Cape, South Africa. The Eastern Cape Province was created in 1994, and include areas from the Xhosa homelands of the Transkei and Ciskei, as well as part of the Cape Province. The Eastern Cape Province is one of the poorest provinces in South Africa [32]. It consists of two metropolitan municipalities, namely, Buffalo City and the Nelson Mandela Bay Metropolitan Municipality as well as six district municipalities; Alfred Nzo, Amathole, Chris Hani, Joe Gqabi, OR Tambo and Sarah Baartman [32]. The study was conducted at the outpatient departments of the six selected primary healthcare centres in Buffalo City Municipality and Amathole Districts, of the Eastern Cape Province, South Africa.

### Study population

The study population was adults with uncontrolled diabetes attending the selected primary healthcare clinics in the Buffalo City Metropolitan Municipality and Amathole health districts, who met the eligibility criteria.

### Inclusion criteria

The inclusion criteria were age 18 years or above; diagnosed with diabetes at least in the last 6 months, currently receiving treatment at the selected clinics, on stable medication for at least three months prior to the recruitment, and with an uncontrolled glycaemic control. In addition to that, the eligible participants must be in possession of a mobile phone, they must be able to retrieve and read SMSs (or have someone who is available to assist with reading the SMS daily) and they must be willing to receive SMSs for the period of the study.

### Exclusion criteria

Participants were excluded from the study if they had health or mental conditions that could interfere with the study or their ability to use or read messages on their mobile phones. Being pregnant or planning to get pregnant within the next six months, and being debilitated or handicapped in such a way that obtaining anthropometric measurements could be challenging were the other exclusion criteria.

Eligible participants were recruited sequentially at the selected health facilities on the clinic days. The recruitment process commenced on the first of July and lasted until the end of August, 2018. The intervention was performed between September 2018 and February 2019, while post-intervention data were collected between March and April 2019.

### Sample size calculation

The previous reported mean HBA1c in the setting was 10.6% [33] and standard care for diabetes using metformin has been reported to reduce HbA1c by 1% (11 mmol/L) [34]. Assuming the intervention adds an extra 0.5% (5.5 mmol/L) and considering a standard deviation of 1 and an alpha error level of 5%, the two-tailed calculation gives a power of 90% with only 84 participants in each arm of the study [29]. A 20% loss of participants to follow-up was anticipated, and a sample of 108 participants was required in each group.

### Randomisation

**Fig 1:**
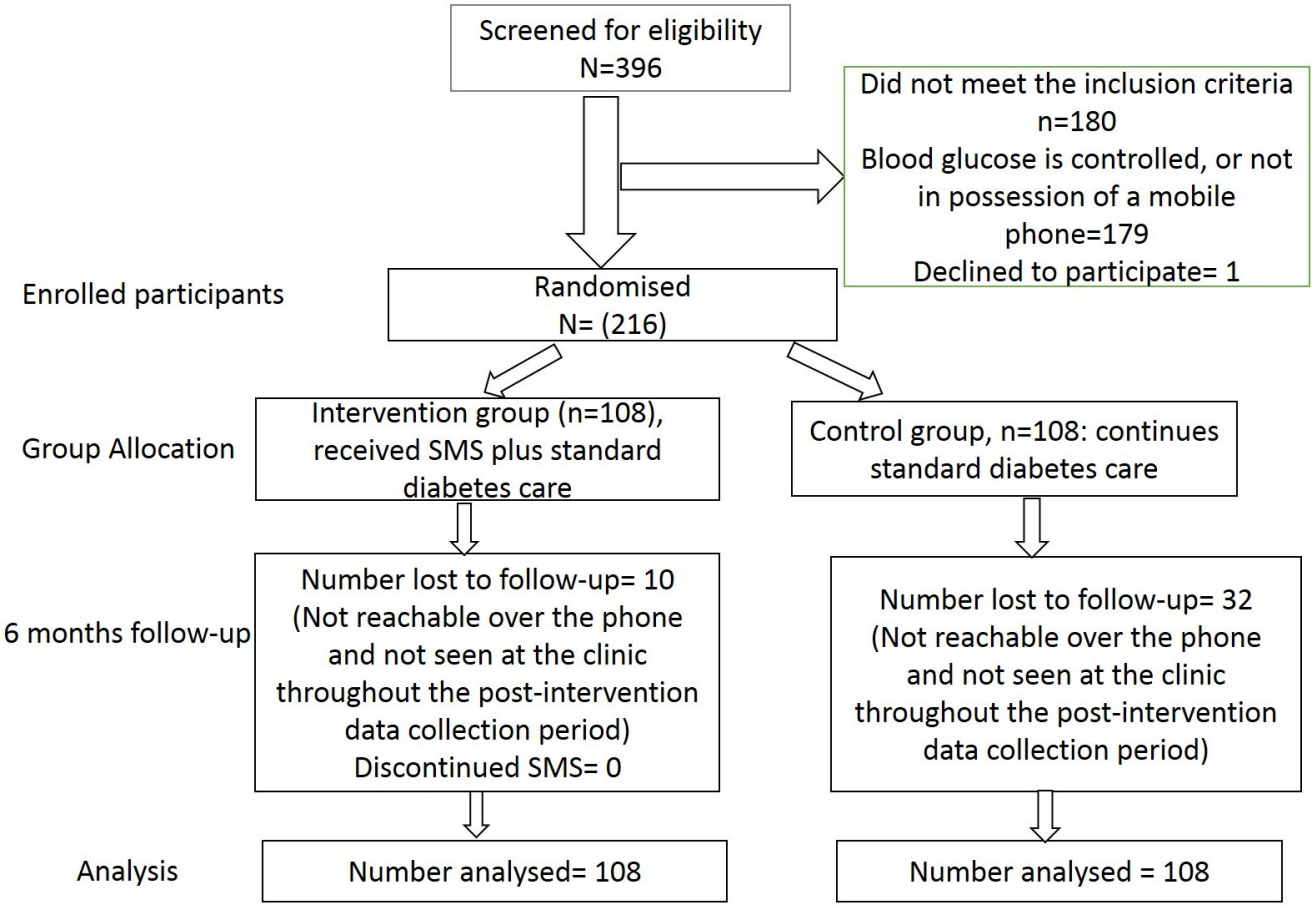
Flowchart of study participants.

The guideline for the management of diabetes is the same across all the primary health care facilities in South Africa [33], thus all clinics were considered eligible; although the quality of care might vary across various health facilities as a result of available infrastructures as well asavailable human resources and the experiences of the care providers. Two of the eight health districts were conveniently selected, from which six clinics were randomly selected. Demographic and other basic information were obtained from the selected clinics, in order to screen for eligible participants. From the sample size calculation, 108 participants were required in each arm of the study, therefore, 36 participants were required from each of the six selected clinics. The 36 participants were randomly selected from the list of eligible participants from each clinic, adjusting for age and mean duration of diabetes. Baseline data were obtained from the randomly selected participants, after which the individuals were randomly assigned to either the intervention or the control arm using their assigned identification number, following a simple randomisation technique, with a 1:1 allocation ratio. Participants in the intervention arm were then contacted to ascertain their preferences in terms of the language of communication (either the locally spoken isiXhosa language or English), time of receiving the SMSs, the name and contact of a next of kin or the available support person. The flowchart for the recruitment, randomisation, allocation and retention of study participants is shown in Fig 1.

### Blinding

The study statistician involved in the randomisation was blinded to all identifying information to avoid bias. Due to the nature of the study, it was impossible for the research staff conducting the SMS intervention as well as the participants in the intervention arm to be blinded to the intervention. However, participants in the control arm were blinded to the intervention. The participants involved in the intervention were privately contacted after randomisation to remind them of the intervention process and aim. The primary outcome, random blood glucose, as an objective measure, and all other measures were also blinded to the treatment allocation.

### SMS development

The principal investigator and her supervisor was supported by a family physician and a nurse in the development of the contents of the SMSs that were sent to the participants. The team followed the Society for Endocrinology, Metabolism and Diabetes of South Africa (SEMDSA) guideline for the management of diabetes, the health education materials from the National Diabetes Education Programme and some sample messages from previous studies which were documented to be efficient [23,35]. The overall health care needs of the participants were not neglected. In addition to that, some gaps in diabetes management in the study setting, as identified in the baseline data also influenced the SMS content. Finally, the opinions of the clinicians and other healthcare workers involved in the management of the patients were put into consideration. The SMS was developed in English and translated to isiXhosa by a professional translator. Both versions of the message were then pre-tested by sending them out to various people with similar characteristics as the study participants, including those with little or no level of education, to ascertain whether they found the message to be easy, simple and clear. Modifications were made using the various feedbacks received. The integrated behaviour change framework further guided the intervention [36]. The Integrated Behaviour Change framework adopted in this study describes the approach and processes which can be utilised to influence health behaviors. The Integrated Theory of Behaviour change proposes that the initiation and continual engagement in health behaviour change (physical activities, compliance with dietary recommendations, adherence to medication use and appointment compliance) can be facilitated by improving condition-specific health beliefs and knowledge, self-regulation skills and ability (goal setting, self-monitoring, reflective thinking, decision making, planning and plan enactment) and social facilitation (support from healthcare centre, support group, SMS, health education). This proximal change, in terms of improved health behaviours could lead to improvement in long-term outcomes related to health, such as blood pressure and glucose control and weight loss. Some examples of the sent text messages are presented in Table 1.

**Table 1:** Examples of SMS contents.

CORE MESSAGES
Control of your blood glucose level require you to eat good food, do exercise and regularly use your pills/insulin as prescribed. Your nurse, dietician and doctor can assist you.
You are the most important member of your healthcare team because you are the one who manage your diabetes day by day
It is important to know your blood glucose level overtime because you do not want your blood sugar to get too high.
Do you know if your sugar is normal or close to normal, you have less chances of developing heart problems, stroke, eye problems and kidney problems? The answer is YES!!!
**HEALTHY EATING**
Eating healthy diet is an important aspect of your diabetes management. It will help in controlling your blood glucose level.
Ensure you make a diabetes meal plan with help of your nurse.
Choose food such as fruits, vegetables, whole grains, bread, cereals, low-fat or skimmed milk and cheese.
Water is the best drink. Choose water rather than juice, regular soda, Twizza drink or coke
Avoid using too much margarine, butter, mayo or salad dressing
**STRESS AND MOOD MANAGEMENT MESSAGE**
Hello [name]. Too much stress can increase your blood sugar. Make sure you have fun and do something you enjoy today. This will help you reduce stress and improve how you feel.
Are you feeling down? If yes, ask help from a friend, family member, clergy, counsellor or your nurse today.
**REMINDERS**
Have you taken your pills today?
Hello [Name]. Did you check your sugar level today or recently?
Hi [Name]. Tomorrow is your next appointment visit to your nurse, do not forget. Going for your appointment helps you and your nurse/doctor manage your diabetes better.
**FOOT CARE**
Check your feet regularly for blisters, red spots or swelling
Looking after your feet will help you prevent foot problems in the future
**EXERCISE**
Set a goal to become more active most days of the week. Start slow by taking 10 minutes’ walk, three times a day.
Stay at or get healthy weight using your planned diets and doing more exercise
**SMOKING AND ALCOHOL**
Avoid taking alcohol in order to better control your diabetes level
Hi [Name]. Good management of your diabetes includes not smoking. Talk to your nurse about how they can help.
**GENERAL**
Brush your teeth daily and floss to keep your mouth, teeth and gums healthy
Report any changes you observe in your health to your nurse/doctor.

One SMS was sent daily, including weekends, and every participant received an average of 184 SMSs for the duration of the study. Core messages which provided a general motivation and educative messages on diabetes were sent thrice a week and messages specifically focusing on dietary aspects were sent twice a week. Messages selected randomly from the various other sections were sent once a week. In this setting, appointments were fixed for every 28 days, thus, individualised reminders for appointments were sent once a month, based on the date of the next appointment provided by the patients. In addition, the few patients who admitted to taking alcohol and smoking, received messages regarding alcohol use and smoking once every weekend. The SMSs were sent through an online bulk SMS platform named Zoomconnect. The platform allows several messages to be sent at once and scheduled when needed. The platform also provided information on the delivery status of the messages (Details of the SMS contents are provided in **S1 File**).

### Assessment of outcome measures

The assessment of the outcome measures was conducted at baseline and at six months after randomisation. At baseline, demographic data and self-reported measures were obtained using a validated questionnaire[34]. Clinical measures were obtained from the available clinical records while the assessment of blood glucose level and other measures followed standard procedure.

The primary outcome measure was a change in the mean morning random sugar, which was measured using an Accu-chek glucose monitoring device. The choice of assessing the random blood glucose was made after preliminary investigations at the various primary healthcare clinics, which indicated that the majority of the patients with diabetes only get to check their HbA1c level once in a year. A similar report was documented by Daramola’s [37] study conducted amongst people living with diabetes in another province in South Africa. As such, relying on the HbA1c level as a measure for making clinical judgement regarding glycaemic control may be unrealistic in this setting and most especially at this level of healthcare where a large number of these patients are being managed. Several studies have long correlated fasting and random blood sugar with HbA1c [38–40]. Although it is not the gold standard for measuring glycaemic control, it could, however, be an alternative measure in resource-poor setting as this, where obtaining the HbA1c level still remains challenging.

Demographic information and clinical history included age, gender, level of education, employment status, income, duration of illness, presence of other comorbidity and medication use.

Other secondary measures were co-morbid outcomes (hypertension and obesity), obtained through blood pressure measurement and anthropometric measurements (body weight and body mass index). Additionally, the acceptability of the SMS intervention was assessed using the participants’ feedback obtained through sets of validated questions [23]. Lastly, the feasibility of the study was determined by the recruitment and retention rates. Further details of the study intervention is provided in the study protocol **S2 File** [41].

### Study intervention procedure

Both the intervention and control groups proceeded with their usual care including all medical visits, tests and diabetes support programmes. In addition, the intervention group received the SMS at an agreed time of the day, according to their needs, care plan and goals. Participants also received motivational and support messages, advice on lifestyle behaviours like diets, physical activity, smoking cessation, medication and appointment reminders. Participants in the control arm only continued with their usual diabetes care at their clinics.

### Ethics considerations

The University of Fort Hare Research Ethics Committee granted the ethical approval for this study. Approval was also sought from the Eastern Cape Department of Health, which included health districts as well as the clinic managers. Verbal and written informed consent was obtained from the participants, before the commencement of the study, after thorough explanation of the research purpose and aims. Rights to anonymity and confidentiality were ensured throughout the study and the participants consented to referral to further care in case of detection of abnormal findings.

### Data analysis

Statistical analysis followed the Intention-to-Treat principle. Descriptive statistics were used to summarise the demographic and baseline characteristics. Continuous variables were summarised as numbers of observed values, means, standard deviation, minimum and maximum. Categorical variables were described as frequency and percentages. Chi-square and Fisher’s exact test were used to assess the difference between groups for categorical baseline variables. For continuous variables, analysis of variance was used to assess the difference in the baseline characteristics of the study participants between the intervention and the control group. The effect of the intervention on the primary outcome between the two groups and at the two periods was assessed using the random effects mixed-model analysis. Scaled Identity covariance structure was used as it produced the least AIC level, with minimal study parameters. Adjustments were made for the type of diabetes, and the baseline outcomes.

Linear regression was used to assess the effect of the intervention on secondary outcome measures between the two groups and two periods. The assumption underlying the analysis of missing variables was that the data were missed completely at random. Missing data were imputed for both the primary and secondary variables using the mean of the variables assessed. A total of 42 participants were lost to follow-up, 10 in the intervention arm and 32 in the control arm, and their missing values were imputed. Sensitivity analyses were performed on assumptions that missing data were not missed at random and the worst-case scenario. All statistical tests were two-sided at 5% significance level. A p-value of <0.05 was considered statistically significant. The Statistical Package for Social Sciences (SPSS) version 23 was used for data analysis (SPSS Inc., Chicago, IL, USA).

## Results and discussion

Of the 216 participants, 108 were in the intervention arm and 108 in the control arm. For both the intervention and the control group, the majority of the participants were females (83.30% vs 85.20%). More than half of the study participants had grades 8–10 (high school) level of education (58.30% vs 58.30%), and the majority had no form of employment (78.70%; 88.90%). The age of the participants ranged from 18 to 87 years. Overall, the mean age of the participants was 60.64 (SD± 11.58) years while the mean income was 108.45 (SD ± 120.20) USD (Table 2).

**Table 2:** Demographic characteristics of study participants by study groups.

Variables	Intervention n (%)	Control n (%)	p-value
**Gender**			
Male	18 (16.70)	16 (14.80)	0.426
Female	90 (83.30)	92 (85.20)	
**Level of education**			
No formal schooling	3 (2.80)	2 (1.90)	0.965
Grade 1–7	39 (36.10)	41 (38.00)	
Grade 8–12	63 (58.30)	63 (58.30)	
Tertiary	1 (0.90)	1 (0.90)	
Post-graduate	2 (1.90)	1 (0.90)	
**Marital status**			
Never married	16 (15.20)	31 (29.00)	0.018
Married	55 (52.40)	47 (43.90)	
Divorced	6 (5.70)	2 (1.90)	
Widowed	28 (26.70)	23 (21.50)	
**Employment status**			
Government employee	2 (1.90)	0 (0.00)	0.209
Non-government employee	7 (6.50)	3 (2.80)	
Self-employed	5 (4.60)	2 (1.90)	
Student	0 (0.00)	1 (0.90)	
Retired	9 (8.30)	6 (5.60)	
Unemployed	85 (78.70)	96 (88.90)	
**Average current monthly Income (Rand)**			
0–1500	39 (37.50)	24 (24.50)	0.032
1501–14200	65 (62.50)	74 (75.50)	

n = Frequency. For intervention group, n = 108; while for control group, n = 108

For both the intervention and the control group, the majority of the participants had Type 2 diabetes (97.20% vs 90.70%), were being treated with oral pills (76.90% vs 74.10%), had concomitant hypertension, (80.60% vs 85.50%) and were receiving treatment for hypertension (75.00% vs 86.80%). Only a small percentage of the participants had no health comorbidity; 27.80% and 16.70% for the intervention and control groups, respectively.

The mean duration of diabetes was 9.06 (SD ± 7.38) years, while that of diabetes treatment was 8.81 (SD ± 7.20) years.

The average random blood glucose level was 14.29 (±4.39) mmol/L for those in the intervention group and 14.39 (±3.41) mmol/L for those in the control arm. There was no significant difference in all the secondary clinical measures at baseline (Table 3).

**Table 3:** Other clinical characteristics of the patients.

Clinical outcomes	Intervention Mean (±SD)	Control Mean (SD)	p-value
Weight (Kg)	83.76 (15.30)	82.09 (17.24)	0.451
Waist circumference (Cm)	98.52 (20.21)	100.23 (15.67)	0.487
Hip circumference (Cm)	109.88 (18.98)	111.69 (21.43)	0.513
Systolic blood pressure (mmHg)	144.28 (21.15)	146.26 (23.84)	0.519
Diastolic blood pressure (mmHg)	82.28 (10.25)	82.75 (15.07)	0.793
Random blood glucose (mmol/L)	14.29 (4.39)	14.39 (3.41)	0.851
Body mass index (Kgm^-2^)	32.21 (5.63)	32.14 (7.16)	0.933

### Impact of the daily text-messaging on glycaemic control

The mean difference in the blood glucose from baseline to six months post-intervention for the intervention group was– 1.58 (SD ± 5.29), while that of the control group was– 1.95 (SD ± 4.69). The mean difference in the change in blood sugar between the two groups from baseline to post-intervention was 0.51 (-0.80 to 1.82), with no significant difference (p = 0.441). After adjusting for baseline blood glucose level, diabetes and treatment type, the mean difference was 0.26 (-0.81 to 1.32), with no significant difference (p = 0.634). The intervention did not have a significant effect on glycaemic status.

Both the intervention and the control group showed slight improvements in the secondary outcomes with no significant difference. There was no significant difference in the mean change in weight, 0.02 (-1.84 to 2.92; p = 0.654), body mass index, 0.03 (-0.54 to 1.24; p = 0.439), systolic blood pressure, -0.03 (-4.98 to 2.93; p = 0.610) and diastolic blood pressure, -0.04 (-2.68 to 1.40; p = 0.535).

### Acceptability and feasibility of the text messaging intervention

Regarding the acceptability of the text messaging (Table 4), the majority of the participants (n = 98; 90.74%) who completed the post-intervention survey indicated the helpfulness of the SMS. Of the 98 participants, 43% maintained that the SMS provided more information about their health and the required diet for health promotion. Also, 24.7% stated that the SMS was a form of reminder to take their medication, while 15.5% said that the SMS not only provided more information, but also served as reminders to take their medication, and was a source of motivation.

**Table 4:** Acceptability of the text messaging intervention.

Variables	Frequency (n)	Percentage (%)
**Did you receive the daily SMS?**		
Yes	98	100.0
No	0	100.0
**Do you think the SMS was helpful?**		
Yes	98	100.0
No	0	0.0
**In what way did the SMS help you?**		
It gives me more information about my health and more especially about my required diets	42	43.3
Reminds me to take my medication and go for my appointments	24	24.7
It motivates me	3	3.1
Reminds me to take my medication, and taught me about the required diet	13	13.4
It reminds me to use my medications, teaches me about the required diets and helps me to stay motivated	15	15.5
**Did the SMS stress you in any way?**		
Yes	1	1.0
No	97	99.0
**Are you satisfied with the timing of the SMS?**		
Yes	96	98.0
No	2	2.0
**If we decide to continue, would you like to continue?**		
Yes	94	95.9
No	4	4.1

When asked about the timing of the SMS delivery, almost all of the participants (98%) were satisfied with the timing of the SMS delivery. Nearly all of the participants (95.9%) declared their readiness to continue receiving the SMS even after the completion of the study should the researcher decide to continue with the SMS.

With regards to the feasibility of th study, out of the 108 participants who participated in the SMS intervention, 91% completed the study. Each of the study participants received a daily SMS throughout the six months, averaging to 180 SMSs per participant.

## Discussion

This study assessed the impact of daily text messaging in addition to standard care on improving glycaemic status and secondary clinical outcomes amongst individuals with diabetes in resource-poor settings in the Eastern Cape Province, South Africa. Sub-optimal glycaemic control is a significant threat to the health of individuals living with diabetes and contributes significantly to the development of microvascular and macrovascular complications [42–44]. At six months post-intervention, both the participants who received the SMS and those who did not receive the SMS, showed improvement in their blood glucose level without any significant difference, even after adjusting for diabetes type and baseline outcome. A similar finding amongst a small sample of patients (90 patients in both arms) in India, indicated a decline in the blood glucose level of participants in the control and intervention groups without any significant difference in the mean change between both groups. However, contrary to this present study, there was more decline among those in the intervention arm [45]. Kollman et al. [46] also documented no significant improvement in fasting blood sugar level following an SMS intervention among patients with Type 1 diabetes in Austria. Other studies assessing the impact of text messaging on glycaemic control mostly used the HbA1c test as a measure of glycaemic control and they reported mixed findings. Some studies showed statistically significant improvement in glycaemic control [23,25,26,35], while, others reported no statistically significant improvement in glycaemic control [46–48].

Although the use of text messaging as an adjunct to clinical care could be of help to patients and could improve health outcomes, it is imperative to consider the interplay of several factors. As explained by Arora et al. [47], the majority of the SMS interventions that have documented improvement in glycaemic control amongst the participants focused more on interventions using bi-directional text messages. The authors further stated that SMS interventions anchored on facilitating continual linkage of the patients to their health care providers or physicians in relation to the communicated blood sugar readings tended to yield a significant improvement in glycaemic control [49]. A similar finding was reported by Quinn et al. [26] who adopted the use of bi-directional messages augmented by enhanced clinical care. Sometimes, the patients’ attitude also influences the outcome. For instance, Capozza et al. [48] indicated that even though participants had the opportunity to send their concerns to the care providers, only a few obliged. Perhaps, the absence of the extra measures which might not have been feasible in this study setting, could explain the non-significant result obtained.

It is well established that various factors underpin glycaemic control which ranges from adherence to the recommended therapy, adequate knowledge, positive self-management behaviour and self-efficacy, as well as the quality of care rendered [9,50–51]. As such, a multi-faceted approach is required to foster improved health outcomes. Notably, the current study was conducted among patients with uncontrolled diabetes and with a low socioeconomic status. Low socioeconomic status, particularly a low level of income and low literacy level are associated with a low level of adherence and poorer health outcomes, including sub-optimal glycaemic control [11, 52–54]. Besides, the factors underlying the poor glycaemic status among this cohort of participants are not clear. Probably, text messaging alone had minimal or no effect on the variables influencing the poor glycaemic status of the participants in this present study.

This study further investigated the impact of SMS intervention on secondary clinical outcomes. There was no significant difference in the mean difference in weight reduction between the two groups. This is in line with various reviews on the impact of the SMS on weight reduction where the text messaging usually result in no significant improvement in weight status [22,55]. Consistent with the findings from other studies, there was no significant difference regarding the impact of SMS on body mass index [56–60]. The two major contributors to weight loss, namely healthy dietary practices and exercise are rarely practised or practised at a low level among the study participants. The highlighted reasons were a lack of financial resources, a lack of time and poor health conditions and these did not improve significantly, even after the intervention. Therefore, the observed non-improvement in weight and body mass index is not surprising. This could also explain the non-significant improvement in glycaemic status. As much as the text messaging can inform the patients about the required and expected healthy behaviours, little can be achieved in bringing about the desirable change without the necessary resources or finances.

Likewise, there was no significant improvement in the blood pressure levels of the participants, both the systolic and the diastolic. Several studies have shown similar findings [8, 26, 61–64]. Contrarily, a few other studies showed a significant improvement in blood pressure following mHealth interventions [57,60,65]. The non-significant change in blood pressure could stem from the recorded insignificant change in blood sugar level and vice versa, since a positive linear association often exists between the two factors. This could also be aggravated by persistent unhealthy lifestyle behaviours, particularly, dietary practices and physical inactivity. In addition, the poor socio-economic level might have also contributed to this problem [11, 52–54].

### Acceptability of text messaging

The acceptability of the mHealth intervention was further explored among the patients. As shown in the participants’ responses, all the participants stated that the SMS intervention was helpful. Some of the listed benefits of the SMS were an improvement in knowledge, motivation and functioning as a reminder. Generally, there appears to be a high rate of acceptability of text messaging among patients with diabetes. Several studies have supported this notion [47,48, 66–68]. Mostly, the acceptability of the text messaging intervention among patients with diabetes has been associated with its ease of use [59,60,63]. Conversely, a mHealth intervention which involved a non-user-friendly web-based interface showed a low rate of acceptability [57].

### Feasibility of text messaging

Out of the 108 participants who took part in the SMS intervention, 91% completed the study. Some of the participants might have lost their contact details, while others might have relocated elsewhere or transferred to another level of healthcare. Though the message was unidirectional, some participants still responded to some of the questions and sent some concerns through to the investigator. For instance, the message “Have you taken your pills today?” triggered responses from the participants with many responding, “Yes”. Feasibility has often been defined in previous studies as the ability to complete the study intervention and many of the previously published articles demonstrated the feasibility of text messaging as a tool to improve diabetes care, even amongst youths [69–71].

The SMS intervention can be described as an acceptable intervention and a feasible and acceptable adjunct to standard care for diabetes management. However, its efficacy in terms of improving health outcomes needs careful consideration, especially among people in the low socio-economic stratum, people receiving care at low-resource settings (areas with inadequate resources such as funds to cover healthcare or inadequate equipment and supplies and fewer trained personnel) and those attending primary healthcare clinics where quality of care might not be optimal. Though it has been widely shown to have a potential for bringing about improvement, this, however, requires further actions and interventions.

### Strengths and limitations

Several limitations of this study should be considered when interpreting the findings. The main limitation was the use of random blood glucose rather than the HbA1c as a measure of glycaemic status, which might not be optimal. The sample size was calculated based on the initial plan of assessing HbA1c, though, the sample size was also appropriate for capillary blood glucose measurement. HbA1c is an expensive measure and often unavailable or seldom done in resource-poor settings and primary healthcare level, as this study setting. Consequently, using HbA1c as a gold-standard for glycaemic status might not be a feasible measure. There was an attempt to bridge this gap by assessing the average of the previous three to six blood glucose readings, however, not all the patients had their blood glucose measured at every clinic visit. Although participants were instructed to fast for at least 8 hours before the blood testing, there was no way of ascertaining if they actually did, hence, the term random. Also, only a few of the clinics in the selected districts and only two of the eight districts in the province were covered, thus, we cannot generalise the findings to the entire province or districts. Recruitment of the study participants was very challenging because of the lapses in the healthcare records system and we were only able to recruit a very few participants living with Type 1 diabetes. Finally, there was approximately 19% loss to follow-up which might have introduced bias, however, a sensitivity analysis was performed and it affirmed the assumption that the study participants were missed completely at random and the result did not differ when tested, based on various other assumptions.

Notwithstanding the limitations of the study, the study clearly demonstrated a high level of acceptability and feasibility of the SMS intervention and a low level of efficacy among the participants in this study. The true experimental design employed was a significant strength. The use of a multi-centre approach added further credence to the study. The use of an objective measure for primary data, and the use of validated tools are additional strengths. Finally, the finding of this study could serve as a reference point for other related studies in the province, and even in the South African context.

## Conclusions

Similar to previous reports, the use of SMS is an acceptable and feasible measure and serves as an adjunct to standard clinical care in the promotion of health amongst patients living with diabetes in this study setting. Although there was a little improvement, the efficacy of unidirectional text messaging in promoting glycaemic control and improving other clinical variables in this study setting is still doubtful.

## Supporting information

S1 File SMS contents. (PDF)

S2 File Study protocol. (DOCX)

S3 File Study dataset. (SAV)

S4 File Research questionnaire. (DOCX)

S1 Table Consort checklist. (DOCX)
