# Supplementary material for: Efficacy, acceptability and feasibility of daily text-messaging in promoting glycaemic control and other clinical outcomes in a low-resource setting of South Africa: A randomised controlled trial
Source: PLoS One. 2019 Nov 27;14(11):e0224791. doi: 10.1371/journal.pone.0224791 (PMC6881007; doi:10.1371/journal.pone.0224791)
Supplement: S1 File — (PDF) [file pone.0224791.s001.pdf]

SMS CONTENTS

CORE MESSAGES

1. Control of your glucose level require you to eat good food, do exercise, and use your pills. Your nurse, dietician and doctor can assist you.

   Ukulawuleka kweswekile yakho kudinga ukuba utye ukutya okunesondlo, uzilolonge kwaye usebenzise ipilisi zakho ngendlela.
   UGqirha noMongikazi nabo baluncedo kuwe.

   Ngokuthi iswekile yakho ibesendaweni yayo entle yitya ukutya okunesondlo, wenze imisebenzi yomzimba, uqhubekeka ngokutya okuya egazini. UGqirha noMongikazi nabo baluncedo kuwe.

2. Taking care of yourself and diabetes can help you to feel good today and in the future

   Ngokuthi uzihoye wena neswekile yakho, ingakunceda uzive ungcono ngamalanga onke.

3. Do you know when your sugar is close to normal, you are likely to have more energy, be less tired and thirsty, urinate less often, have fewer skin and bladder infections? The answer is YES!!!

   Uyayazi iswekile yakho isondele ekubeni ibengcono? Ungangumntu onomdla wokusebenza, unganxanwa oko okanye udinwe okoko, ungabingumntu ochama oko, amathuba akho ohlaselwa yingxaki yesikhumbo somzimba kunye nengxaki nesinye sakho.
   Impendulo ngu Ewe!!!

4. Do you know when your sugar is close to normal, you have less chances of developing heart problems/stroke, eye problem and kidney problem? The answer is YES!!!

   Uyayaz xa iswekile yakho isendaweni yayo? Ubuyazi namathuba ohlaselwa zizifozentliziyo/stroke angangqongophala? Ingxaki yamehlo kunye nezintso?
   Impendulo ngu Ewe.

5. You are the most important member of your healthcare team because you are the one who manage your diabetes day by day

Ungoyena mntu ojongene nempilo yakho kuba nguwe ozokujongana neswekile yakho ntsuku zonke.

6. It is important for you to know your blood sugar level overtime. You do not want your blood sugar level to get too high.

   Kubalulekile utshekishe iswekile yakho qho. Akufunekanga iswekile yakho ibephezulu okanye inyuke.

7. High levels of blood sugar can harm your heart, kidney, feet, eyes and blood vessels

   Ukwenyuka kweswekile ingakwenzela ubenesifo sentliziyo, izintso, inyawo ezikuphatha kabuhlugu, amehlo,negazi elingacocekanga.

8. Tell your nurse you would like to know your HbA1c level, a test that helps you to know your average blood sugar over the past three months. It is different from the one you do at each clinic visit.

   Xelela umongikazi ukuba ufuna ukwazi iswekile yakho ingakanani emzimbeni, ungqomfwe umnwe kujongwe ingakanani emzimbeni emvakwenyangana ezintathu. Ihlukile le udlangoyenziwe eclinic.

9. If your blood pressure gets too high, it makes your heart work harder and can cause heart attack, stroke, damage your kidneys and eyes.

   Ukuba ipressure yakho iphezulu kakhulu, yenza intliziyo yakho isebenze kanzima ingakunikezala iingxaki zentliziyo, izistroke, okanye ukonakala kwezintso namehlo.

10. Ask your nurse what your blood pressure goal is and work towards that by taking your blood pressure pills as prescribed, doing exercise and eating good diet.

    Buza kumongikazi wakho umfufuzo kujongwe awunyukelwanga yihigh-high, usebenzisana nokutya ipilisi zakho zehigh-high njengoba uxelelwe, nokwenza umsebenzi usebenzisa umzimba nokutya ngendlela.

11. Ask your nurse what your blood cholesterol is and work towards that by taking your prescribed pills as you were told and eating good diet.

Buza umongikazi igazi lakho linjani, uqhubekeke ngokutya ngendlela iipilisi zakho ngendlela oxelelwe ngalo uqhubeke ngokutya ngendlela ukutya okunesondlo.

12. Excessive weight does not allow your blood sugar to be controlled. Do exercise such as walking or gardening almost daily and eat good diet always to keep your weight under check, even if you feel better.

    Ukubanomzimba omkhulu ayikuchazeli ukuba iswekile yakho isendaweni yayo. Qhubeka uwusebenzisa umzimba wakho ngosebenza,njengo hamba umgama omde okanye usebenze igadi yonke imihla okanye ithuba uthe walifumana nakhona ungcedisana nendlela yokutya uqhubekeke nokujonga umzimba wakho.

13. Take your pills as prescribed by your nurse/doctor daily even when you feel good.

    Thatha ipilisi zakho uzitye ngendlela ekuthiwa zitye ngayo ngumongikazi okanye ugqirha qho ngosuku nokuba uziva ungcono.

14. Take your diabetes pills always as prescribed even when you feel good.

    Thatha iipilisi zakho njengoba uxelelwe nokuba sosemandleni.

15. Keep track of your blood sugar, check and record in a book and always talk about it with your healthcare provider.

    Qhubekeka ujonga iswekile yakho, mayibhalwe encwadini uqhubeke nokuthetha nabancedisi bomongikazi.

16. Check your blood pressure and keep a record of it.

    Qhubeka uyijonga iswekile yakho uyibhalelwe phantsi encwadini.

17. Blood testing is the best test for measuring blood sugar. Testing your sugar in the urine is not the same as testing your sugar in the blood.

    Ukuhlolela iswekile egaini kubalulekile ngaphezu koyihlolela emcamweni.

18. Attending your diabetes appointment does not stop you from having complications but it can help you know in time when you do and can help you to quickly treat or manage it.

ukuya rhoqo kwicheck up yakho yeswekile ayinqandi ukuba ungavelelwa zingxaki kodwa yenza uhlale ulumkile kwangexesha nageyiphi into enothi yenzeke.

SMOKING AND ALCOHOL CESSATION MESSAGE

1. Hi [name]. Good management of your diabetes and your future health includes not smoking. Talk to your nurse about how they can help.

   Molo [igama]. Ingqubo elungileyo yokuhoyana neswekile yakho kunye nobomi bakho ayihambelani nokutshaya. Thetha nomongikazi wakho ngalemeko ngokuba bazakunceda.

2. HI [name]. Avoid taking alcohol in order to better control your glucose level and future health.

   Molo. Musa ukuqhubekeka notywala ukuze impilo yakho ibesendaweni entle kunye nobomi bakho.

3. For better control of your blood sugar, you need to stop smoking. Ask your nurse for help to quit.

   Yeka utshaya. Buza uncedo kuyekwa njani.

4. Alcohol is an empty form of energy without any nutritional value. Excessive amounts of alcohol will lead to weight gain.

   Utywala siselo esingalunganga sikunikeze amandla kungekho zakha mzimba. Ngokuthi usele umzimba wakho ubamkhulu uvuleke. Umntu angasela iglass enye ngosuku(Omama). Ko Tata (iglass ezimbini ngosuku). Isiphuzo ngasinye yi=125ml wine, 340 ml lite beer, 1 tot= 25ml spirit.

HEALTHY EATING MESSAGES

1. Eating healthy diet is an important aspect of your diabetes management. It will help in controlling your blood glucose level.

   Ngokutya ngendlela kubalulekile kwisekile yakho. Izakunceda ekubeni iswekile yakho ibesendaweni yayo entle.

2. Make a diabetes meal plan with help from your nurse.

   Bhala phantsi izinto okumele uzitya ngokuthu uncediswe ngumongikazi wakho.

3. Eat foods with more fibre such as whole grain cereals, brown bread, crackers, brown rice or whole-wheat pasta.

Yitya ukutya okuneFibre izinto ezifana nengqolowa ipapa, isonka esenziwe ngengqolowa.irice ebrown kunye nepsta eyenziwe ngengqolowa.

4. Choose foods such as fruits, vegetables, whole grains, bread, cereals, low-fat or skimmed milk and cheese.

   Khetha ukutya neziqhamo,imifuno, isonka, ipapa, izinto ezingatyebanga, kusetyenziswe ibisi olungatyebanga necheese.

5. Drink water instead of juice, regular soda, twizza or coke.

   Sela amanzi kunokusela ijuice okanye isoda, twizza okanye icoke.

6. When eating your meal, fill half of your plate with vegetables and fruits, one quarter with lean portion such as beans or chicken or turkey without the skin, one quarter with whole grain such as brown rice and whole wheat pasta.

   Xa usitya ukutya wakho, into mayibeninzi mayibeyimufuno utye neziqhamo, ikota enye yongeza ekondlekeni iibeans nenkukhu okanye itheki uzisuse isikhumba, ikota enye yebrown rice okanye ipasta yengqolowa.

7. Fats are energy-dense and consuming high levels of fat can lead to weight gain or being overweight.

   Ukutyeba kuqaba ungabinamandla nakhona kunyusa amazing aphezulu okutyeba.

8. Use healthy cooking methods: grill, bake, steam, poach, microwave, pressure cook or boil. You can also use cooking spray or a non-stick pan.

   Sebenzisa indlela elungileyo okutya, ukuyiqhotsa, ekubhakeni, ekuyibiliseni,ekufudumezeni, indllela yokutya kubile. Ungasisebenzisa ispray sokupheka.

9. Avoid using too much margarine, butter, mayo or salad dressing.

   Zama ukuyeka usebenzisa into eqatywayo like irama, mayonnaise ne salad dressing

10. Eat vegetables that are half your plates or size you can hold in your two hands. Choose from any of these common vegetables: Broccoli, Cucumber, Green beans, Leafy salad greens (including Romaine lettuce), Lettuce, Collards, Turnip greens, Green herbs e.g. Parsley, beetroot, Carrots, Pumpkin, Red peppers, tomato, cabbage, Cauliflower, Eggplant (brinjal), Mushrooms and Onions.

    Itya ivege ehlaza kakhulu like lettuce, green beans, broccoli, cauliflower njalo-njalo

11. Cook your vegetables in a healthy way and try to add only a little butter, sugar, cream, cheese sauce or oil.

    Pheka imifuno yakho ngendlela elungileyo uzame ugalela nentwana yerama,iswekile kancinci,icream,icheese sauce okanye uthi chatha intwana yamafutha.

12. Fill a quarter of your plate or a size of your closed fist with whole-grain starchy foods like Brown/Wild rice, Couscous/Quinoa, Legumes/Soya/Hummus, Mealie meal (pap, phutu, porridge), Oats/oat brand, Pearled barley, Pearled/bulgur wheat, Sorghum, whole grain crackers/bread/cereal, Whole grain/seeded breed or Whole wheat pasta, butternut and potatoes.

    Ikota yeplate yakho makubekhona istarch ezinjenge Brown/Wild rice, uMili-mili(papa), iOats/oats beand, ibhathanathi kunye netapile.

13. Avoid white bread or rolls, samp, white rice as they are lower in fibre and can increase your blood glucose.

    Sukusebenzisa isonka esimhlophe okanye irolls, umgqusho, irice emhlophe ngokuba zinesinikamdla osezantsi nakhona zinyusa iswekile.

14. Eat you mealie meal or porridge cold or allow it to cool down to lower the effect on blood sugar.

    Yitya imili-mili okanye uwusebenzise epapeni.yilinde iphole izingenyusi iswekile yakho

15. Fill a quarter of your plate or size of the palm of your hand with protein such as White fish e.g. hake, Salmon, pilchards, sardines, snoek, mackerel and herring, Lean poultry chicken and turkey (remove the skin), Red meat - beef, lamb, pork with visible fat removed (eat limited amounts), dried beans, lentils, chickpeas, Eggs, Tofu, Cottage cheese. Hard cheese can be included in small amounts. Opt for lower fat options e.g. mozzarella cheese.

    Itya izinto ezinje nge whitefish umzekelo hake, salmon, pilchard, sardines, snoek, mackerel and herring, lean poutry chicken and turkey, ususe iskin, ungazitya nazo izinto ezifana ne red meat kodwa usise iskin nakuzo uxobule namanqatha or skin and uzame ukuzitya kancinci zona, idriend beans namaqada ungawatya kodwa in small portions.

    Enye ikota esityeni sakho makubekhonaifish emhlophe umzekelo iHake, iSalmon,ipilchards, isardines,usnoek,imackerel kunye nerring, ibanenkukhu uyisuse ufele,inyamana ebomvu,inyamama yehagu nayo isusiwe inqatha, ibeans zome, amaqanda,chickpeas, Tofu, Cottage cheese. Icheese etyebileyo ungayisebenzisa kodwa ingabininzi umzekelo imozzarella cheese.

16. Foods such as chocolates, biscuits etc. that are labelled “Suitable for diabetics” OR “Sugar Free” OR “No sugar Added” are not recommended. Remember, sugar-free does not mean carbohydrate and calorie free.

Ukutya okufana nezinto ezisweet njenge chocolates,biscuits nezinye zibhaliwe ukuba akukho swekile efakiweyo. Kumbula ezi zithi akukho swekile zincedesana unganyukelwa yiswekile.

17. Eating too much salt in your diet can cause high blood pressure (hypertension) which can cause damage to the kidneys, heart, brain and eyes.

    Xa utya kakhulu ityuwa ingakwenza ubenehigh-high leyo imosha izintso zakho.

18. Sugar substitutes may be used instead of sugar to sweeten foods and drinks. Sugar substitutes contain very few kilojoules and will therefore not affect your blood glucose levels. Example is the sweetex given at the clinic.

    Iswkile ezi zizipilisi ezincinci ungazisebenzisa ekutyeni okanye kwisiphuzo sakho. Intwana yeswekile yezapilisi ziyanceda nakhona aziyichaphazeli iswekile yakho.Umzekelo iSweetex ezi nizinikezwa eclinic.

19. Water is the best drink and it is recommended. Add fruit slices (e.g. strawberries), cucumber slices, lemon juice or mint leaves to your water to vary the taste. Avoid any sugary drinks (e.g. sugar-containing fizzy drinks, cordials, iced tea).

    Amanzi abalulekile uwasele. Neziqhamo kunyanzelekile uyitye njenge (strawberries), icucumber uyisike, ilamoni juice, okanye amagqabi emint uwagalele emanzini akho ukuze ibenencasa. Sukusebenzisa iziphuzo ezineswekile(umzekelo iifizzy drinks, cordials okanye iezibandayo eziqinileyo.

20. Avoid fruit juice.

    Sukuyisela ujuice

21. Avoid hot drinks that contain sugar e.g. hot chocolate, Horlicks and Milo.

    Sukusebenzisa iziphuzo ezishushu ezineswekile, njenge hotchocolate, horlicks, nemilo.

22. You may include tea and coffee in your diet. Avoid adding sugar to your hot drinks.

    Ungayiphunga itea necoffee kwidiet yakho, kodwa ungayifaki iswekile

23. Fruits are very high in vitamins, minerals and fibre just like vegetables. Fruit do contain carbohydrates so keep that in mind with your meal plan. 1 portion of fruit = size of a tennis ball (e.g. small apple, orange or pear), 2 golf balls (e.g. 2 plums), small banana, handful of grapes, ½ cup of cut-up fruit, 30g dried fruit (± 2 pieces),

    Ifruit nevege ibalulekile ngoba ine vitamins, minerals and fibre enintsi zam ukutya ifruit enintsi uyixubaxube.

± 125ml 100% fruit juice (with no added sugar). Try to include a variety of different fruits.

24. If you must take yoghurt, ensure it is a plain yoghurt. Avoid sweetened yoghurts.

    Xa uzotya iyoghurt, itya le ibhalwe plain yoghurt.

25. The diet you choose to eat as a diabetic patient is not just good for you only, other people without diabetes can also be encouraged to eat it. The diabetes diet is a healthy diet for most people.

    Indlela ukhetha ngayo ukutya kwakho njengomntu oneswekile iyakho good kuew qha, nabanye abantu abangenaswekile bangakhuthazela ngoba kubalukile nakuwo wonke umntu.

26. A portion of chicken does not have more fat than low-fat milk. Choose food items that are lower in fat.

    I ntwana yenyama yenkukhu unamafuthu amakhulu ngaphezu kobisi iwe low fat.

27. A portion of chicken does not have as much fat as a low fat milk. Even though the milk is said to be low fat, there is still some contents of fat in there. Reduce the intake of food items with fat.

    Intwana yenyama yenkhulu ayinamafuths amaninzi nje nge low fat milk, nangona kuthwa ubisi lu low fat ulugqibelelanga ngoko ke nciphisa izinto ezinamafutha.

28. Rather than fry, you can cook your meat in water, stew or put in the oven.

    Pheka ngamanzi odwa. Njengaxa upheka isityu okanye I suphu okanye pheka kwi oven okanye emlilweni.

29. You are allowed to braai meet with fat like pork, chiken or fish, just remove the fat before braaing.

    Inyama engatyenanga enje ngeyenkhukhu, ihagu okanye ifishi.

30. Water is the best drink. Drink at least 8 glasses of water everyday.

    Ukuba uyasela, sela amanzi ikomityi ezisibhozo ngosuku. Akululgele emzimbeni wakho.

STRESS AND MOOD MANAGEMENT MESSAGE

1. Hello [name]. Too much stress increase your blood sugar. Make sure you have fun and do something you enjoy today. This will help you reduce stress and improve how you feel.

Molo. Ukuba nestress esikhulu, kungusa iswekile yakho yonwaba wenze into ekanwabise, leyonto izakwehlisa istress neswekile.

2. Try deep breathing, gardening, taking a walk, listening to your favorite music or doing your hobby today in order to reduce stress.

   Zama Ukuba nezibilini ezise zatsi, thatha uhamba umgamana onde, umamele unculo owuthandayo, ukuze ungabinaso istress.

3. Are you feeling down? If yes, ask for help from a friend, family member, clergy, counsellor or your nurse today.

   Uziva unomoya uphantsi? Ukuba ewe, cela uncedo kumhlobo wakho okanye komnye umntu kusapho, okanye kubancedi bocinizelelo iwengqondo okanye konongikazi.

REMINDERS

1. Have you taken your pills/insulin today?

   Uzithathile ipilisi okanye insulin namhlanje?

2. Hi [name]. Today is your appointment visit. Do not miss it.

   Molo. Namhlanje lusuku iwakho lokuya eclinic ungalibali.

3. Attending your appointments regularly gives your nurse the opportunity to monitor your condition properly, quickly detect any abnormality and counsel as appropriate. Do not miss your appointments.

   Ukungaziphosi iappointments zakho uye qho kuzo kwenza inurse zikuxilonge kakuhle bakwazi ubona yonke into eyenza kuwe ngexesha, suziphosa iaapoinments zakho.

4. Do you still have your diabetes pills? Or are you running out of it? If you don't have or you are running out of it, go to your clinic for more pills.

   Unazo na ipilisi zeswekile? Uba awunazo iya eclinic uyofumana zona

5. Do you still remember you next appointment date? Do not miss it.

   Idate yakho elandelayo usayikhumbula na? ungayilibali

FOOT CARE MESSAGE

1. Looking after your feet will help you prevent foot problems in the future. Check your feet daily and contact your nurse, doctor or foot doctor if there are any changes.

Ukujonga inyawo zakho rhoqo kuceda ukungabinazifo zenyawo. Dibana noGqirha, uNurse okanye uGqirha wenyawo xakukho umehluko kwinyawo zakho.

2. Check your feet everyday for cuts, blisters, red spots or swelling.

   Jonga inyawo zakho yonke imihla ukwenzela ukwazi uqhabela ukusikeka, imibhala ebomvu okanye ukudumba.

3. Visit your clinic immediately you observe a sore that doesn’t go away.
   Iya eclinic ngokukhawulweza kuzoxilongwa ukudumba okungapheliyo.

4. Do not wear too fitted or tight shoes.

   Suzinxiba izahlangu ezincinci okanye ezikubambayo kakhulu, qhiniseka ukuba inyawo zakho zisoloko zinespace esi free.

EXERCISE

1. Set a goal to become more active most days of the week. Start slow by taking 10 minutes’ walk, three times a day.

   Bhala phantsi icinto ozakuzenza ngosuku , qala kancinci umane uhamba hamba kathathu ngemini.

2. Stay at or get a healthy weight using your meal plan and doing more exercise.

   Hlala kumzimba wakho omhle nusebenzisana nokutya okutyayo.

3. Increase your physical activity pattern. Start slow by taking 10 minutes’ walk, three times a day.

   Yandisa kakhulu nakwi exercises zomzimba. Qala kancinci umane uhamba hamba kathathu ngemini.

4. Exercising regularly can help reduce your blood pressure.

   Ukuzilonga umzimba ngokwenza iexercise rhoqo kunceda ukwehlisa ihigh blood pressure.

OTHERS/GENERAL

1. Brush your teeth daily and floss to keep your mouth, teeth and gums healthy.

   Hlamba amazinyo akho rhoqo yonke imihla ukwenzela umlomo wakho uzobasempilweni.

2. Talk to your nurse/doctor if you have any questions about your diabetes.

Theta nomongikazi, ukuba unembuzo yeswekile yakho komongikazi.

3. Report any changes you observe in your health to your doctor or nurse.

   Chaza ingxaki nemphile yakho ekuphete kakubi.

4. At each visit, be sure to check your blood sugar, blood pressure, weight and foot and review how well you are doing with your diabetes plan.

   Qho undwendwela iclinic, jonga iswekile nepressure ujonge umzimba wakho, uhlolo iwanyawo, iswekile yakho ibesendaweni entle.

5. When your sugar drops too low and you feel symptoms like shaking, trembling, blurry eyes, weakness, dizziness, pale skin, sweating, rapid pulse, hunger, nausea, irritability and feeling confused. Immediately eat or drink something that is high in sugar such as 125ml of regular Coke (not Coke Light) OR a 125ml glass of fruit juice OR 1 Tablespoon jam or honey OR 3 teaspoons sugar in water. This will push your sugar level up quickly (within 10 – 15 minutes).

   Xa iswekile yakho iszantsi kakhulu, umzimba uyashukuma qmehlo ankungu, ukukanomzimba aphantsi, ubedizzy, ubene sikhumba somzimba esicekeceke, ukubile, ukadikwa, ukuziva ulahlakelwa ngumqondo khawalleza utye okanye usele into eneswekile njenge coke ne juice yesiqhamo 125 izakwenguko iswekile yakho.

SMS GROUPS

1 = MORNING, XHOSA
2 = MORNING, ENGLISH
3= AFTERNOON, XHOSA
4 = ANYTIME, XHOSA
5= ANYTIME, ENGLISH
6 = EVENING, XHOSA
