# Supplementary material for: Efficacy, acceptability and feasibility of daily text-messaging in promoting glycaemic control and other clinical outcomes in a low-resource setting of South Africa: A randomised controlled trial
Source: PLoS One. 2019 Nov 27;14(11):e0224791. doi: 10.1371/journal.pone.0224791 (PMC6881007; doi:10.1371/journal.pone.0224791)
Supplement: S2 File — (DOCX) [file pone.0224791.s002.docx]

EFFICACY, ACCEPTABILITY AND FEASIBILITY OF mHEALTH TECHNOLOGY IN PROMOTING ADHERENCE TO ANTI-DIABETIC THERAPY AND GLYCAEMIC CONTROL AMONG DIABETIC PATIENTS IN EASTERN CAPE, SOUTH AFRICA

A research proposal submitted to

DEPARTMENT OF NURSING SCIENCE,

FACULTY OF HEALTH SCIENCES,

UNIVERSITY OF FORT HARE.

By

EYITAYO OMOLARA OWOLABI (#201502792)

Supervisor: Prof DT Goon

April, 2017

1. **INTRODUCTION**

Diabetes mellitus (DM) is an important public health concern, and forms part of the four priority non-communicable diseases (CVD, cancer, and chronic respiratory diseases) targeted by world leaders for special attention (WHO, 2016a:5). The burden associated with diabetes is enormous, thus the 2016 World Health Day was dedicated to diabetes with the slogan, “Beat Diabetes” (WHO, 2016b).

Diabetes mellitus is a costly disease (Seuring, Archangelidi & Suhrcke, 2015:811). It is one of the leading causes of death and disability worldwide (International Diabetes Federation (IDF), 2013:32). The proportion of people living with diabetes is dramatically on the increase (IDF, 2014:2; IDF, 2013:36; Whiting et al., 2011:1) and disturbingly high. In 2014, about 8.3% adults (387 million) were estimated to be living with diabetes globally, with as many as 46.3% undiagnosed (IDF, 2014:2). Globally, mortality associated with diabetes has also been on the increase; rising from 4% to 9% between 2012 and 2014 (WHO, 2014a).

Of course, diabetes mellitus is no longer a disease of predominantly rich nations. It is becoming increasingly prevalent among the poor in developing countries, even among the “poorest of the poor” (Anjana et al., 2011:3; Dasappa et al., 2015:1; Hwang et al., 2012:1). Almost three-quarters of people living with diabetes are currently in low and middle-income countries (LMICs) (IDF, 2014:2; Shen et al., 2016:1). The burden of diabetes in under-resourced areas is complicated by lack of effective and adequately-equipped healthcare systems as well as deficiency in prevention strategies (Margaret Chan in WHO, 2016b).

Over 4 million South Africans are affected by diabetes; about 7% of the population, with as many as 41% of cases still undiagnosed (Health 24, 2016; IDF, 2015). Diabetes is responsible for 6% of the mortality in 2012 in South Africa (Alwan et al., 2011; WHO, 2014b). The epidemic of diabetes mellitus in South Africa is driven by obesity resulting from unhealthy lifestyles (Manyema et al., 2015). The Eastern Cape Province is not exempted from this menace. Owolabi et al. (2016) reported a high prevalence (24%) of diabetes among adults in one of the two largest municipalities (Buffalo City Metropolitan Municipality) in the Eastern Cape Province.

Diabetes mellitus is a disease characterised by an abnormally high blood glucose level (hyperglycaemia) (American Diabetes Association, 2011:1). Uncontrolled hyperglycaemia is often associated with a high risk of developing either microvascular or macrovascular health complications (Cade, 2008:1; Kirkman et al., 2012:3). Microvascular complications of diabetes include diabetic nephropathy, neuropathy and retinopathy, leading to kidney disease, amputation and blindness (Asif, 2014:1; Deshpande, Harris-Hayes & Schootman, 2008:3), while macrovascular complications include coronary artery disease, peripheral arterial disease, and stroke (Chawla, Chawla & Jaggi, 2016:3; Fowler, 2011:1). These complications further impose greater burden on the individual, reduce the quality of life, increase the risk of premature mortality and impact enormous burden on the healthcare system (Nolan, Damm & Prentki, 2011:1).

However, diabetes mellitus is treatable, though cannot be cured, except for the recently proposed surgical intervention, specifically gastric bypass (Eenfeldt, 2015). Various treatments allow most individuals with diabetes to live relatively stable and normal lives (Asif, 2014:1; Diabetes UK, 2017). Intensive therapy for patients with diabetes mellitus delays the onset and as well slows the progression of clinically important complications such as retinopathy, including vision-threatening lesions, nephropathy and neuropathy by a range of 35 or more than 70% (The Diabetes Control and Complications Trial Research Group, 1993).

Blood glucose control indicated by a reduction in the HbA1c level is accompanied by a reduction in the risk of complications (American Diabetes Association, 2011; 9). As low as 1% reduction in HbA1c  has been reported to be associated with a 21% reduction in the risk of any end-point death related to DM, 14% reduction in the risk of developing myocardial infarction as well as 37% reduction in micro-vascular complications (ACCORD Study Group, 2010; Ismail-Beigi et al., 2010:1; Stratton et al., 2000).

However, in spite of the innovative scientific discoveries and treatment modalities, it seems that treatment outcomes of individuals with diabetes are generally suboptimal. Diabetes control in developed countries is below average (Teoh et al., 2010). Also, diabetes control in sub-Saharan Africa (SSA) is generally suboptimal (Camara et al., 2015:3). The prevalence of uncontrolled diabetes range from 62% in Nigeria (Ngwogu et al., 2014), 68% (Congo) (Longo-Mbenza et al., 2008), 70.5% (Saudi Arabia) (Khan et al., 2014), 79.2% (Uganda) (Kibirige et al., 2014) to 82% in Botswana (Mengesha, 2007). Similarly, sub-optimal control of DM has been noted in South Africa (Folb et al., 2015:4; Webb, Rheeder & Van Zyl, 2015:6). The prevalence of sub-optimal control in various provinces of South Africa ranges from 69.3% in Northwest (Kadima & Tumbo, 2013), 81% in Cape Town (Daramola, 2012), 83% in Kwazulu-Natal (Igbojiaku et al., 2013) to 84% in Eastern Cape Province (Adeniyi et al., 2016). This indicates a higher prevalence of uncontrolled diabetes in South Africa, compared to other countries in SSA with Eastern Cape Province, with the highest prevalence in South Africa.

Various reasons have been documented to be responsible for this poor glycaemic control. Such factors include duration of disease (Khattab et al., 2010:1; Nemeh, Yousef & Aysha, 2011:3) and comorbidity (Woldu et al., 2014:3). Other factors include overburdened healthcare facilities, insufficient health education, sub-optimal knowledge and beliefs, poor support structures, unsafe communities and low income (International Diabetes Federation 2016; Kibirige et al., 2014). However, a major causative factor of poorly controlled diabetes among individuals with diabetes is non-adherence to treatment regimen and recommended lifestyle changes for the diabetic patients (Aikens & Piette, 2013:1; Davies et al., 2013:9; de Vries et al., 2014:3; Hamine et al., 2015:1; Jarab et al., 2014:1; Kassahun, Eshetie & Gesesew, 2016:3). There are wide range of documented evidences of non-adherence to treatment among individual with diabetes both globally and nationally (Mann et al., 2009; Krishna & Boren, 2008; Mbuagbaw et al., 2012; Rwegerera, 2014).

Non-adherence to medication among patients has long been recognised as a pressing problem in South African primary healthcare system (Kagee, 2004). Patients are the centre of the healthcare team and the importance of increasing their knowledge base on diabetes has been documented to influence diabetes self-management (Amod et al., 2012; Mandewo et al., 2014; Santos et al., 2015). Viewed in this perspective, there is a need for interventions to enhance patient’s education and improve their self-treatment behaviours and self-management skills. Limited control trials of various interventions have been conducted among diabetic patients in order to determine their effectiveness in boosting patients’ knowledge and self-care behaviours. Such interventions include educational programmes using various media as well as lifestyle interventions (Berg et al., 2009; Mash et al., 2012; Schwellnus et al., 2009). However, there is limited documented interventional research available to address the correlates of uncontrolled diabetes in South Africa.

The use of information and communication technology such as computers, mobile phones, satellite communications, patient’s monitors among others to support health services and information exchange are available (Hamine et al., 2015:1; Wiggins, 2015:88). Of course, mobile technology has the potential to improve the quality, safety and efficiency of healthcare services, and to impact almost every aspect of health sector (Cole-Lewis & Kershaw, 2010; Jemberu, 2013). The World Health Organisation also prioritised the use of new technologies to assist healthcare delivery in resource-limited settings (Joint United Nations Programme on HIV/AIDs, 2005; WHO, 2011). The most widely used technology is the mobile phones, with an evidence of increasing uptake in Africa (Lester et al., 2006; Sinha & Barry, 2011). The use of mobile phones has been shown to have the potential to radically improve healthcare in the most remote and resource-poor environments of the world (Hamsphire et al., 2015:1; Marwaha, 2010). Even before phones became mobile, Hyanes et al. (1996) had already posited that communication effort that would keep the patients engaged in healthcare may be the simplest and most-cost-effective strategy for improving adherence to chronic medications. The use of mobile phones has been documented to improve treatment adherence, appointment compliance and developing support networks for health workers (Hall et al., 2014). Likewise, the use of SMS on the mobile phones is recognized as a means of providing new and innovative opportunities for disease prevention efforts at a low cost (International Diabetes Federation, 2016).

Various randomised controlled trials on the use of SMS has been conducted in various parts of the world including Africa; and found to be cost-effective and associated with an improvement in self-management behaviour, lifestyle changes, medication compliance and improved blood glucose control among the study participants (Carole et al., 2012; Dobson et al., 2016; Ferrer-Roca et al., 2004; Jemberu, 2013; Jha et al., 2015:4; Islam et al., 2014; Mohammed et al., 2014).

There is a rapid increase in the use of mobile phones in South Africa; and are more affordable and available now (Leon, Schneider, Daviaud, 2015:4). Also, in South Africa, successes have been recorded with the use of mHealth technology among patients with various conditions such as hypertension (Leon et al., 2015), sexually transmitted infections (STIs) and HIV/AIDS (de Tolly, Nambaware & Skinny, 2011:1, malaria (Quan et al., 2014:5) and maternal and child health conditions (Baron et al., 2016:4). However, there is little or no documented interventional studies applying the use of mHealth technology strategy for diabetes control among individuals with diabetes (Leon et al. 2015; Lunny et al., 2014). Thus, there is need to carry out interventional studies targeting the use of mHealth among diabetic individuals.

1. **PROBLEM STATEMENT**

Despite the effectiveness of drug therapy in diabetes management, high rate of poor adherence persists among diabetic patients, both globally and nationally (Adisa & Fakeye, 2014; Bagonza et al., 2015; Cramer, 2004; Mann et al., 2009). A surge in the prevalence of diabetes has been documented in South Africa with an increasing disease burden and mortality associated with it (IDF, 2015). South Africa has the second highest prevalence of diabetes in Africa (Guarigata et al., 2013:3). Also, glycaemic control among diabetic patients in South Africa appears to be a challenging task for healthcare professionals (Shilubane, 2010). This is likely to be consequential to the documented poor adherence to medication among diabetic patients attending the primary healthcare facilities (Booysen & Schlemmer, 2015; Kagee, 2004); as intensive medication therapy and compliance with the prescribed medication regimen is a key factor for glycaemic control. Consequently, there is a resultant predisposition to complications development leading to poor quality of life, disability, premature mortality and excessive burden on the individuals; thus placing significant strain on the already overburdened healthcare system.

mHealth is an emerging and a cost-effective measure proven to be effective in improving patient’s self-management behaviours, drug regimen adherence and appointment compliance. However, the effectiveness of mHealth among diabetic patients has rarely been documented in South Africa, and more specifically, in an economically poor region of the Eastern Cape Province. Hence, the need for an interventional study to determine the efficacy of mHealth technology, aside the usual care in promoting glycaemic control and adherence to anti-diabetic therapy among individuals with diabetes in a low-resource setting.

1. **AIM AND OBJECTIVES**

The overall aim of this interventional study is to determine the efficacy, acceptability and feasibility of the mHealth technology in promoting adherence to anti-diabetic therapy and glycaemic control among diabetic patients in low resource areas of the Eastern Cape Province, in order to inform public health direction concerning diabetes control.

1. **OBJECTIVES**

**The specific objectives of the study are:**

- Determine the efficacy and acceptability of mobile phone SMS in promoting glycaemic control among individuals living with diabetes in the Eastern Cape Province.
- Determine the efficacy and acceptability of mobile phone SMS in promoting adherence to medication, physical activity and recommended diets among individuals living with diabetes in the Eastern Cape Province.
- Determine the effectiveness of the use of mobile phone SMS as a clinic appointment reminder or awareness promoter about diabetes and its complications.
- Determine the efficacy of the use of mobile phone SMS in improving diabetes knowledge among individuals living with diabetes in the Eastern Cape Province.
- Determine the healthcare providers’ perspective on the use of mHealth technology in diabetes control in the Eastern Cape Province; and
- Determine the efficacy of text messaging in improving other secondary clinical outcomes (body weight, blood pressure and body mass index).

1. **RESEARCH QUESTIONS**

- Would the use of mobile phone SMS be effective in promoting glycaemic control among individuals living with diabetes?
- Would the use of mobile phone SMS be effective in promoting adherence to medication, physical activity and dietary recommendations among individuals living with diabetes?
- Would the use of mobile phone SMS be effective in promoting diabetes knowledge among individuals living with diabetes?
- How effective is the mobile phone SMS as a clinic appointment reminder and awareness promoter on diabetes and its complications?
- What are the likely barriers to the use of SMS in conveying health information to persons living with diabetes in the Eastern Cape Province?
- How do the healthcare providers perceive mHealth technology as a feasible option in the control of diabetes?

1. **SIGNIFICANCE OF THE STUDY**

Poor adherence to anti-diabetic therapy has been identified as a threat to effective glycaemic control as well as prevention of complications among individuals living with diabetes. Since diabetes is chronic in nature, patient’s involvement in their care as well as improvement in their self-care management behaviour will go a long way in improving their quality of life.

Also, improving adherence to anti-diabetic therapy will play a key role in reducing morbidity and mortality related to diabetes. It is believed that the SMS services have the potential to build awareness about the disease, improve self-management and prevent complications also in resource-limited settings. Since the SMS trials has been documented to be effective in other countries and for certain other diseases, the findings of this study might help to determine if this same measure can be applied to those in the low-resource areas, and if it will yield any positive result. If this intervention proves to be efficient in the current study, large-scale implementation could be undertaken across all the diabetes clinics in the province and in the country at large.

Finally, the findings of this study will help the policy makers to understand the importance of creating healthcare systems that better meets the need of people and develop prevention and management strategies for diabetes and other chronic diseases using innovative mobile phone technologies at the national level.

1. **SCOPE AND DELIMITATION**

The study is delimited to adults who were already diagnosed of diabetes, receiving anti-diabetic therapy and are attending the selected diabetes clinics in the Eastern Cape Province. Variables of interest will be mhealth technology (SMS) and diabetes control among individuals living with diabetes.

1. **DEFINITION OF KEY OPERATIONAL TERMS**

**Efficacy**: It is defined as the ability of the mHealth intervention to produce intended results; that is, enhancing self-management, promotion of medication adherence, and glycaemic control among diabetic individuals.

**Acceptability**: It is defined as the acceptance of the mHealth intervention by the diabetic individuals.

**Feasibility**: It is the ability to carry out the mHealth study among the diabetic individuals.

**Adherence**: In this study, adherence means sticking to the prescribed anti-diabetic therapeutic regimen by diabetic individuals.

**mHealth**: An abbreviation for mobile health. It is the use of mobile devices, specifically, SMS, to support and promote diabetes control.

**Glycaemic control**: It is the regulation and maintenance of blood glucose levels within normal range.

**Therapy**: The treatment and management of diabetes mellitus; including medical and lifestyle management.

**Anti-diabetic therapy:** Administration of drugs, diets and exercise in order to reduce blood glucose level.

1. **SUCCINCT LITERATURE REVIEW**

**9.1 Definitions of Diabetes Mellitus**

Diabetes Australia (2015) defines diabetes as a chronic disease characterised by high blood glucose levels. Diabetes mellitus is a chronic disease resulting from an inherited and /or acquired deficiency of insulin production by the pancreas or the ineffectiveness of the insulin produced. As such, it leads to an increased concentration of glucose in the blood (American Diabetes Association, 2011:1; Da-Vita, 2016; WHO, 2016c). Diabetes mellitus (DM) is viewed as a group of metabolic diseases which are chronic and progressive in nature, characterised by elevated levels of blood glucose resulting from defects in insulin secretion by the pancreas, insulin action or both insulin secretion defects and action (ADA, 2011; WHO, 2016a:5).

**9.2 Classifications of diabetes Mellitus**

There are three major types of diabetes;

Type-1 diabetes: This is a form of diabetes that is most common among children and teens and often referred to as juvenile diabetes mellitus. It is as a result of failure of the body to produce insulin and only 5-10% of diabetes cases are type-1.

Type-2 diabetes: This is also called adult-onset diabetes. It occurs frequently among the adults. It results from deficiency of insulin production in the body or the ineffectiveness of the insulin produced. Majority of the cases of diabetes are type-2.

Gestational Diabetes: This third type of diabetes is usually associated with pregnancy and often disappear with pregnancy (Da-Vita, 2016).

**9.3 Diagnosis of diabetes**

The various methods of diagnosing DM include the use of glycated haemoglobin (HbA1c), oral glucose tolerance, the fasting blood glucose level and the random blood glucose level, all of which can be tested during regular health checkups (ADA, 2015b; John, 2012:2; National Institute of Diabetes and Digestive and Kidney Diseases, 2016; WHO, 2011: 6; WHO, 2006:2).

**9.3.1 Glycated haemoglobin (HbA1c)**

Glycated haemoglobin (HbA1c) is utilised to detect type-2 DM and pre-diabetes. It shows an individual’s average blood glucose level over the past three months without any daily changes. It is considered a convenient method, as it does not require fasting and it is time independent (American Heart Association, 2016; National Health System, 2016; NIDDKD, 2016). This tool has been validated and accepted as a diagnostic tool for diabetes. It was formerly used for monitoring blood glucose (WHO, 2011:6).

**9.3.2 Oral glucose tolerance test (OGTT)**

The oral glucose tolerance test can be used for diagnosing diabetes, pre-diabetes, and gestational diabetes. It has been confirmed to be more sensitive than the fasting plasma glucose test, but is less convenient to carry out. It requires the individual being tested to fast for eight hours, after which a blood sample is drawn for testing, with another sample drawn two hours after the ingestion of any liquid containing 75 grams of glucose (American Heart Association, 2016; National Health System, 2016; NIDDKD, 2016).

**9.3.3 Fasting plasma glucose (FPG)**

Fasting plasma glucose (FPG) is another tool used in the diagnosis of diabetes and pre-diabetes. It a more convenient and cost-effective tool than the oral glucose tolerance test. It requires the individual to fast for eight hours prior to the test, which is conducted, preferably, in the morning (American Heart Association, 2016; National Health System, 2016; NIDDKD, 2016).

**9.3.4 Fasting capillary glucose (FCG)**

Fasting capillary glucose is another tool for diagnosing pre-diabetes and diabetes. It differs from the fasting plasma glucose in that a capillary blood sample is used for conducting the test, rather than the plasma. In epidemiological surveys, fasting capillary glucose (FCG) is used in place of fasting plasma glucose because of its convenience and cost-effectiveness (Zhao et al., 2013). It is also a validated tool (Bortheiry, Maleri & Franco, 1994; Kruijshoop et al., 2004) ).

**9.4 Burden of diabetes**

Diabetes mellitus is a public health concern and forms part of the four priority NCDs (the others being CVD, cancer, and chronic respiratory diseases) targeted by world leaders for special attention (WHO, 2016a:5). The proportion of people living with diabetes has increased over the years. In 2011, 366 million people were reported to have diabetes worldwide, with a large percentage of them residing in LMICs (Whiting et al., 2011:1). In 2013, 382 million people were living with diabetes; about one in every 10 adults, a figure that was projected to increase to 592 million by 2035. A higher rate is expected among men (14 million more than women), and urban dwellers (110 million more than rural dwellers globally and 59 million more than rural dwellers in LMICs). The Middle East and Northern Africa had the highest prevalence of diabetes (10.3%), followed by North America and the Caribbean (9.1%), with the lowest prevalence being in Africa (5.9%) (International Diabetes Federation (IDF), 2013:36). In 2014, the number of people living with diabetes globally rose to 387 million (8.3%), that is, one of every 12 people, with as many as 46.3% undiagnosed. Prevalence was highest in the western Pacific region and lowest in the Africa region (IDF, 2014:2).

Diabetes mellitus is the fourth leading cause of mortality in most high-income countries (HICs) (IDF, 2013:32). In 2012, 1.5 million deaths among adults were attributable to diabetes, which is about 8.4% of the global all-cause mortality. An additional 2.2 million deaths from pre-diabetes, totalling 3.7 million abnormal blood glucose level-related deaths in the year 2012, which were mostly premature in nature (IDF, 2013: 56). There is evidence of increased prevalence and burden of diabetes in developed countries such as the USA (Mason, 2011; CDC, 2011), the UK (Hex et al., 2012:1;) and other European countries—Spain, Germany, Italy and France (Kanavos et al., 2012: 32).

The evolving epidemic of DM in sub-Saharan Africa (SSA) is triggered by urbanisation and westernisation which has led to the adoption of sedentary lifestyles and unhealthy dietary practices (Hall et al., 2011:1; IDF, 2013:7). This, in turn, has become a public health and socio-demographic concern in SSA in the face of scarce resources (Mbanya et al., 2010:1). A surge in the epidemic of DM is already taking place in South Africa, indicating looming trouble for its health system, resources and economy (Dr. Larry Distiller in Health 24, 2016). In 2015, 2.28 million South Africans were reported to be affected by diabetes; about 7% of the population. As many as 41% of cases were still undiagnosed (IDF, 2015). The prevalence of pre-diabetes was 8.5% (Health 24, 2016). The prevalence of diabetes varies between different races in South Africa, with the highest prevalence recorded among the South African Indian population (11.13%) as a result of their genetic predisposition to DM, followed by coloured South Africans (8.10%), blacks (5.8%) and whites (4%) (Health 24, 2016). Another study in a rural area of South Africa, the Agincourt district, found a prevalence of 3.5% and 1.9% for diabetes and pre-diabetes with no significant gender variation in diabetes prevalence (Motala et al., 2008:3).

Diabetes is a social and economic issue. Direct cost of diabetes ranges from $242 on out-patient expenditure in Mexico to $11,917 for cost of DM in USA while indirect cost ranged from $45 in Pakistan to $16,917 for the Bahamas. However, majority of the cost of diabetes in LMICs are out-of pocket expenses against that of HICs and this affect people in LMICs grossly and directly (Seuring et al. , 2015:811). The burden of diabetes in South Africa is associated with a cost of $949 per person (IDF, 2015).

**9.5 Diabetes and behavioural risk factors**

Diabetes is closely linked with behavioural risk factors such as physical activity, overweight and obesity, smoking and alcohol consumption. Smoking is an established cause of diabetes. Smokers have a 30% – 40% increased chance of developing type-2 DM. It is linked to diabetes in both individuals with normoglycaemia and those with hyperglycaemia (Willi, Bondenmann & Ghali, 2007:1). Smoking has been reported to be responsible for increased blood sugar levels. It also impedes the control of blood sugar, thus increasing the chances of developing complications related to diabetes, such as kidney disease, blindness, and amputation; the risk increases as the rate of smoking increases (Park, 2011; USDHHS, 2014:19; Yang et al., 2002:2). The pathophysiologic route through which smoking stimulates diabetes is the stimulation of insulin resistance, low-grade inflammation, endothelial dysfunction as well as influencing the development of other lifestyle risk factors (Fagard & Nilsson, 2009:4).

Moderate alcohol consumption was documented to be associated with an increase in insulin sensitivity and glycaemic control among individuals with diabetes and a reduction in HbA1c concentrations among non-diabetics, specifically among women (Lun et al., 2013:5; Schrieks et al., 2015:6). It protects both men and women from type-2 diabetes at a moderate level. However, heavy consumption of alcohol is deleterious to health (Baliunas et al., 2009:7). Blomster et al. (2014:3) also showed a reduction in risk of cardiovascular disease and all-cause mortality in relation to moderate alcohol consumption among individuals with diabetes mellitus.

According to the National Institute of Diabetes and Digestive and Kidney Diseases (NIDDKD) (2014: 5), physical activity promotes healthy weight and helps to prevent and improve diabetes. It helps in increasing the sensitivity to and activity of insulin, thus keeping blood sugar within normal range (WHO, 2010). Both moderate and high-level physical activity have been associated with a reduced chance of developing diabetes and a reduced death rate among those who already have it (Jonker et al., 2006:2).

Overweight and obesity have been identified as key determinants of the incidence of diabetes (Pinto & Beltrán-sánchez, 2015:1). Of significant importance is the distribution of fat (abdominal obesity) and its deleterious health effect in the development of insulin resistance and glucose metabolism, which predisposes to diabetes (World Heart Federation, 2016).

The needs of diabetic patients are not only limited to adequate glycaemic control but also correspond with preventing complications; disability limitation and rehabilitation. Healthy lifestyle behaviour, in addition to blood glucose monitoring and compliance with medication regimen as well as healthy coping skills have been identified to be key in predicting good outcomes of diabetes management (Shrisvastava, Shrivastava & Ramasamy, 2013:1).

**9.6 Factors responsible for poor adherence to diabetes therapy**

Poor adherence to diabetes therapy has a grievous impact on glycaemic control (Aikens & Piette, 2013:1). There are several potential reasons for poor adherence to diabetes therapy and such factors cluster in diabetic patients (Garcia-Perez et al., 2013). Garcia-Perez et al (2013) identified age, polytherapy, duration of disease, cost and psychological factors as determinants of poor adherence to diabetes therapy. Sankar et al (2015:1) however reported low-income, irregular blood monitoring and inadequate health education by health professionals as the potential causes of poor adherence to therapy among diabetic individuals. Jackson et al (2015:1) identified similar factors, in addition to depression, as determinants of poor adherence. In spite the slight variation in the factors responsible for poor adherence to diabetes medication, the impact of knowledge of diabetes on therapeutic adherence among its sufferers was emphasised by many authors (Garcia-Perez et al., 2013; Jackson et al., 2015; Sankar et al., 2015).

The importance of knowledge of diabetes in the management of diabetes cannot be over-emphasised (Iwueze, 2007). Knowledge has been identified as the greatest weapon in the fight against diabetes (Khan et al., 2015:1). Knowledge of diabetes, self-care management and its complications among diabetic patients has been reported to influence health-seeking behaviours and better management of diabetes and its complications (Uchenna et al., 2009:5). Education of diabetic individuals has long been reported to bring about an improvement in HBA1c (Norris et al., 2002) and being advocated for by various international organisations. Sankar et al. (2015) also suggested the need to prioritise interventions focusing on enhancing patients’ knowledge in promoting medication adherence. This was further reinforced by Carratala-Munuera et al. (2015), who showed significant improvement in adherence to therapy following better patient information.

mHealth technology is a vital tool to improve the quality, safety and efficiency of healthcare services (Jemberu, 2013). It has been shown to bring about significant improvement in knowledge and management of diabetes among diabetic individuals as well as improved clinical outcomes (Carole, 2012; Hall et al., 2014; Marwaha 2010). Several studies have employed the use of mHealth technology in disseminating information in order to boost patients’ knowledge (Hamsphire et al., 2015:1; Marwaha, 2016) and it has proven effective in promoting knowledge and adherence to medications in several conditions (Leon et al., 2015; Quan et al., 2016), including diabetes (Carole et al., 2012; Dobson et al., 2016).

**10. Theoretical Framework**

**10.1 The Integrated Theory of Behaviour change**

The Integrated Theory of Behaviour change is based on the assumption that behaviour change is a dynamic and iterative process (Ryan, 1998)). This theory suggests that health behaviour change through engagement in self-management behaviours can be enhanced by fostering knowledge and beliefs, increasing self-regulation skills and abilities and enhancing social facilitation (Institute of Medicine, 2001). Engagement in self-management behaviour is often related to greater likelihood of achieving the desired result. It further shows that desire and motivation are pre-requisites to change, and self-regulation facilitates the progress (Lorig, Ritter, Plant, 2005). It also highlights that positive social influences power one’s interest and willingness just as positive relationship helps to support and sustain change. It is also assumed that person-centred interventions, which are often aimed at increasing knowledge, beliefs, skills and abilities are more effective than standardized interventions in promoting health behaviour change. According to the theory, there will be more likelihood for an individual to engage in the recommended health behaviours if they are provided with information, embrace health beliefs consistent with behaviours if they develop self-regulation abilities and experience social facilitation that positively influences and support them (Barlow, Sturt, Hearnshaw, 2002).

**Knowledge and Beliefs**

Knowledge of diabetes

Personal perception

Self-efficacy

Outcome expectancy

**Outcome**

**Proximal Distal**

**Engagement in self-management behaviour** (physical activity, compliance with clinic visits, no smoking, no alcohol consumption, adherence to therapy)

**Self-regulation skill and ability**

Goal setting

Self-monitoring and reflective thinking

Decision making

Planning and plan enactment

Self-evaluation

**Improved Health Status**

Reduced HbA1c

Improved blood pressure

Weight loss

**Social facilitation**

Influence

Support (health facilities, policies, support group, health education, SMS)

**Figure 1:** Integrated Behaviour Change model

10.2 **Application of the theory to the study**

Education and engagement in healthy lifestyle behaviour has been identified as key components in the improvement of health and management of chronic conditions. Glycaemic control and prevention of complications associated with diabetes requires diabetic individuals to engage in health promoting behaviours, and comply with therapeutic regimen. Based on the integrated theory of behaviour change, diabetic individuals will be assessed for their knowledge on diabetes, factors associated with adherence and diabetes control as well as their health beliefs related to diabetes. Participants’ glycaemic level, blood pressure, weight and behavioural characteristics will be assessed. Based on the findings of the initial assessment, participants will receive text messages tailored to their needs with the aim of increasing their knowledge, enhance their self-efficacy and outcome expectations in relation to their glycaemic level. Feedbacks from participants will also be expected as they will be encouraged to forward all queries or issues to the cell number that will be provided. At the end of the intervention, the measure of the expected outcome; self-management behaviours, improvement in lifestyle behaviours and ultimately clinical outcomes; weight, blood pressure, HbA1c, physical activity, smoking and alcohol use will be assessed.

**11. RESEARCH DESIGN**

This study will adopt a quantitative, descriptive, interventional approach to determine the efficacy, acceptability and feasibility of the mHealth technology in promoting adherence to anti-diabetic therapy and glycaemic control among diabetic patients in the Eastern Cape Province. This will be an interventional study, utilising a randomised controlled trial to compare six months standard care with a mobile phone-based SMS intervention among diabetic patients receiving treatment.

**12. STUDY SETTING AND POPULATION**

The study will be conducted at diabetes clinics in four randomly selected districts in the Eastern Cape Province, South Africa. The Eastern Cape Province was created in 1994, and include areas from the Xhosa homelands of the Transkei and Ciskei, as well as part of the Cape Province. The Eastern Cape Province is one of the poorest provinces in South Africa (Business Tech, 2016; StatsSA, 2011). The Eastern Cape Province is made up of two metropolitan municipalities; Buffalo City Metropolitan Municipality and the Nelson Mandela Bay Metropolitan Municipality and six district municipalities. The six district municipalities are; Alfred Nzo, Amathole, Chris Hani, Joe Gqabi, OR Tambo and Sarah Bartman districts municipalities (Wikipedia, 2017).

**13. TARGET POPULATION**

The target population will be diabetic patients receiving treatment at the primary healthcare centres in the Eastern Cape Province.

### 13.1 Inclusion Criteria

### Participants will be included in the study if they are 18 years and above; diagnosed of diabetes mellitus at least within 6 months; have a HBA1c level of >7.0%, or a random morning glucoe > 10mmol/l, receiving treatment, on a stable treatmnet for 6 months, dose for one month and is in possession of a mobile phone; and able to read a SMS or have an available relative willing to assist in reading the SMS.

### 13.2 Exclusion criteria

Participants will be excluded if they have psychiatric disorders, pregnant, debilitated, handicapped in any form such that obtaining anthropometric measurements will be difficult or with any cognitive impairment or any form of impairment that will hinder the use of cell phones.

**14. SAMPLE SIZE**

Standard care for diabetes using metformin has been reported to reduce HbA1c by 1% and the intervention adds an extra 0.5% (Amod et al., 2012; Pal et al., 2013:22). Assuming a standard deviation of 1 and an alpha error level of 5%, the two-tailed calculation gives a power of over 90% with only 90 participants in each of the control and intervention group. If 20% loss of participants to follow-up is considered (Islam et al., 2014), a total of 108 participants is required in each group.

**15. RANDOMISATION AND BLINDING**

In order to avoid sharing of messages among diabetic patients, different districts will be used for the control and the intervention. The guideline for the management of diabetes is similar across all the primary health centres in South Africa (Amod et al., 2012), thus, all the primary health care centres are eligible. Of all the eight municipalities/districts, two were conveniently selected. In each selected district, the diabetes clinics were assessed for available facilities such as human resources, HbA1c testing, available diabetes support programmes. The information were obtained from the various facilities or unit heads as deemed appropriate. The obtained information were scored and used to stratify the diabetes clinics into two levels; average and low resourced clinics. All the clinics were assigned an identification number which were hidden from the study statistician. One average-resourced and two low-resourced clinics were selected from the two selected districts, summing up to 6 clinics; 4 low resourced and 2 high resourced clinics. From the clinics selected, demographic and other basic information were obtained to screen for eligibility. From the sample size calculation, 108 participants are required in both arms of the study, therefore, 36 participants are required from each of the six selected clinics. After the collection of the baseline data, randomisation will take place, where individuals are randomly assigned to either the intervention or the control arm using a simple randomisation technique with a 1:1 allocation ratio using the assigned ID numbers. The study statistician will randomly select 36 participants from the list of eligible participants from each clinic, adjusting for age and mean duration of diabetes. Participants in the intervention arm will then be contacted to ascertain their preferences in terms of the preferred language of communication; either the locally spoken Xhosa language or English, preferred time of receiving SMSs, name and contact of next of kin or the available support person.

The treatment allocation will be concealed until the point of randomisation. However, due to the nature of the study, the selected districts for the intervention will be made aware of the intervention. Likewise, it will not be possible for research staff conducting the SMS intervention to be blinded to the intervention. However, the primary outcome, random fasting sugar will be blinded to treatment allocation since it is an objective measure.

**16. DATA COLLECTION AND INTERVENTION**

Both intervention and the control groups will continue with their usual care including all medical visits, tests, and diabetes support programmes. In addition, the intervention group will receive SMS at an agreed time and days of the week tailored according to their needs, goals and their care plan. Participants will as well receive motivational and support messages and they can opt to receiving additional messages on topics such as lifestyle intervention; diets, physical activity and smoking cessation. Where appropriate to their care, patients will also receive reminders to check their blood glucose levels, insulin or drug use. Contacts to be reached in case of any query will be provided at the end of the message. Call records will be kept.

Data will be collected at baseline, and six months after the intervention. Baseline data will include demographic characteristics including duration of diabetes, behavioural factors (smoking, physical inactivity, alcohol use), anthropometric measurements (weight, height, waist and hip circumferences) and biochemical measurements (random blood sugar). At baseline, the demographic characteristics, particularly, age, and duration of diabetes as well as mean blood sugar will be balanced between both arms.

**17. PRIMARY OUTCOME MEASURE**

**17.1 Glycaemic control**

The primary outcome measure is a change in glycaemic control from baseline to six months, measured as mean random blood glucose level. This will be assessed using a point-of care device which analyses a drop of fingertip capillary blood.

**18. SECONDARY OUTCOME MEASURES**

The secondary outcome measures will be assessed at three and six months namely;

- Knowledge on diabetes which will be obtained with the validated Michigan Diabetes Knowledge Test Questionnaire-2 (Fitzgerard et al., 2016).
- Medication adherence, using a pre-validated self-developed medication adherence questionnaire.
- Clinic attendance, using clinic registers.
- Quality of life, using the EQ-5D self-perceived quality of life questionnaire (EURO-QOL Group, 2009).
- Barriers to SMS intervention and the healthcare providers’ perception about SMS use using a self-designed questionnaire.
- Clinical (hypertension, overweight/obesity) and behavioural outcomes (Smoking, alcohol use, and physical activity) will be measured using the WHO modified STEPwise approach.
- Factors affecting adherence to diabetes therapy using a self-designed questionnaire

**19. SMS DEVELOPMENT AND DISPERSAL**

The messages will be developed by a team involving the principal investigator, supervisor, general physicians, endocrinologists and a nurse. Also, health education materials from the National Diabetes Education Programme will be used. Once the messages have been developed, they will be sent to several individuals including those with no formal education in order to ascertain its simplicity. The questions will be developed based on the principles of the behavioural change theory (Leventhal & Cameron, 1987). SMS will be dispersed to all participants in the intervention arm on a daily basis over the period of study at an agreed time of the day.

**20**. **ETHICAL CONSIDERATION**

The ethical approval for this study will be sought for from the University of Fort Hare Research Ethics Committee. Approval will also be sought from the Eastern Cape and the sub-districts Departments of health. Lastly, approval will be sought from the clinic managers. Also, verbal and written informed consent will be obtained from the participants before the commencement of the study.

**21. DATA ANALYSIS**

All treatment evaluations will be performed on the principle of intention-to treat (ITT), using the observed data collected from all randomised participants. Comparison of the baseline and the subsequent readings of the HbA1c will be determined. Primary outcome will be analysed with the chi-square. Descriptive statistics will be used to summarise the demographic and baseline characteristics. Continuous variables will be summarised as numbers of observed values, means, standard deviation, median, minimum and maximum. Categorical variables will be described as frequency and percentage. Linear regression model will be used to test the effect of intervention on the primary outcome between the two groups. All statistical tests will be two-sided at 5% significance level. The Statistical Package for Social Sciences (SPSS) version 22 will be used for all statistical analysis.

**22. CHAPTER OUTLINES**

Chapter one of the study will orientate the reader about the study. Chapter two will focus on relevant literature pertaining broadly, to the use of mHealth technology and diabetes control. Chapter three will include the research methodology. Chapter four will focus on the results, represented in tables, graphs and charts as well as the discussion of findings. Summary, conclusion and recommendations will be presented in chapter five.

**23. RESULTS DISSEMINATION**

The thesis will be kept in the school’s library. Likewise, results will be presented at seminars and conferences. Lastly, the findings of this study will be published in accredited peer reviewed journals.

**24. TIME FRAME**

The proposed timeline for the study is illustrated in Table 1.

**Table 1**: Timeline for the research project

|  | Months | | | | | | | | | | | | | | | | | |  |
| --- | --- | --- | --- | --- | --- | --- | --- | --- | --- | --- | --- | --- | --- | --- | --- | --- | --- | --- | --- |
| Activity | April 2017 | May, 2017 | June, 2017 | July, 2017 | Aug 2017 | Sept 2017 | Oct. 2017 | Nov. 2017 | Dec. 2017 | Jan, 2018 | Feb, 2018 | Mar, 2018 | April 2018 | May 2018 | June, 2018 | July 2018 | Aug., 2018 | Sept..2018 | May 2019 |
| Writing of research proposal |  |  |  |  |  |  |  |  |  |  |  |  |  |  |  |  |  |  |  |
| Literature review |  |  |  |  |  |  |  |  |  |  |  |  |  |  |  |  |  |  |  |
| Presentation of proposal to the faculty |  |  |  |  |  |  |  |  |  |  |  |  |  |  |  |  |  |  |  |
| Registration with senate higher degree committee |  |  |  |  |  |  |  |  |  |  |  |  |  |  |  |  |  |  |  |
| Registration with the provincial Department of Health |  |  |  |  |  |  |  |  |  |  |  |  |  |  |  |  |  |  |  |
| Registration with the Districts Department of Health |  |  |  |  |  |  |  |  |  |  |  |  |  |  |  |  |  |  |  |
| Formulation of the SMS by the team |  |  |  |  |  |  |  |  |  |  |  |  |  |  |  |  |  |  |  |
| Training of research assistant |  |  |  |  |  |  |  |  |  |  |  |  |  |  |  |  |  |  |  |
| Baseline Data collection |  |  |  |  |  |  |  |  |  |  |  |  |  |  |  |  |  |  |  |
| Data capturing |  |  |  |  |  |  |  |  |  |  |  |  |  |  |  |  |  |  |  |
| Collection of data after 3 months |  |  |  |  |  |  |  |  |  |  |  |  |  |  |  |  |  |  |  |
| Literature review continues |  |  |  |  |  |  |  |  |  |  |  |  |  |  |  |  |  |  |  |
| Collection of data after 6 months |  |  |  |  |  |  |  |  |  |  |  |  |  |  |  |  |  |  |  |
| Data capturing |  |  |  |  |  |  |  |  |  |  |  |  |  |  |  |  |  |  |  |
| Data Analysis |  |  |  |  |  |  |  |  |  |  |  |  |  |  |  |  |  |  |  |
| Compilation of reports |  |  |  |  |  |  |  |  |  |  |  |  |  |  |  |  |  |  |  |
| Submission for examination |  |  |  |  |  |  |  |  |  |  |  |  |  |  |  |  |  |  |  |
| Final corrections |  |  |  |  |  |  |  |  |  |  |  |  |  |  |  |  |  |  |  |
| Graduation |  |  |  |  |  |  |  |  |  |  |  |  |  |  |  |  |  |  |  |

**25. RESEARCH BUDGET**

| **Activity** | **Estimated Cost** |
| --- | --- |
| Printing of questionnaires for the baseline and final data collection | R10000 |
| Printing of informed consent | R8000 |
| Employment of Research Assistants for data collection at baseline and at 6 months | R40,000 |
| Procurement of instruments (Laptop, data storage device, Point of care device for sugar, test-stripes for sugar) Blood pressure apparatus, height and weight scale) | R30000 |
| Employment of Nurses involved in dispatching SMS and follow-up for the period of 6 months | R30,000 |
| Procurement of recharge cards for disseminating SMS for the period of study | R40000 |
| Data Capturing at baseline, 3 months and 6 months | R20,000 |
| Logistics(accommodation during data collection, transport, miscellaneous) | R22,000 |
| Data Analysis | R15,000 |
| Language Editing | R10000 |
| Printing and binding of dissertation | R7,000 |
| Presentation of results at Conferences | R15,000 |
| Printing and binding of dissertation | R3000 |
| Total | R250,000 |

26. **REFERENCES**

Adeniyi, O.V., Yogeswaran, P., Longo-Mbenza, B. & Goon, D.T. (2016). Uncontrolled hypertension and its determinants in patients with concomitant type 2 diabetes mellitus (t2dm) in rural South Africa. *PloS ONE*, 11(3): e0150033.

Adisa, R. & Fakeye, T.O. (2014). Treatment non-adherence among patients with poorly controlled type 2 diabetes in ambulatory care settings in southwestern Nigeria. *African Health Sciences*, 14(1):1–10.

Aikens, J.E. & Piette, J.D. (2012). Longitudinal association between medication adherence and glycaemic control in type 2 diabetes. *Diabetic Medicine*, 30: 338–345.

Alwan, A., Armstrong, T., Cowan, M. & Riley, L. (2011). N*on-communicable Diseases Country Proﬁles 2011*. World Health Organization, Geneva, Switzerland: 1-207.

American Diabetes Association (2011). Standards of Medical Care in Diabetes-2011. *Diabetes Care*, 34(1):S11–S61.

American Diabetes Association (2015). *Definition of Diabetes. Definition of diagnosis*. Available at: http://www.diabetes.org/diabetes-basics/diagnosis/ [Accessed June 22, 2015].

American Diabetes Association (2003). Treatment of hypertension in adults with diabetes. *Diabetes Care*, 26(Supplement 1): 80–82.

American Heart Association (2016). Symptoms, monitoring and diagnosis of Diabetes. Diabetes. Available at: http://www.heart.org/HEARTORG/Conditions/Diabetes/SymptomsDiagnosisMonitoringofDiabetes/Symptoms-Diagnosis-Monitoring-of-Diabetes_UCM_002035_Article.jsp#.V1UOo_l97IV [Accessed June 6, 2016].

Amod, A., Motala, A., Levitt, N., Berg, J., Young, M., Grobler, N., Dave, J., Distiller, L., Ganie, Y., Grobler, N., Heelbrunn, A., Huddle, K., Janse Van Rensburg, G., Jivan, D., Joshi, P., Khutsoane, D., Levitt, N., May, W., Mollentze, W., Motala, A., Paruk, I., Pirie, F., Raal, F., Rauff van Zyl, D. & Young, M (2012). Type 2 diabetes guideline. *Journal of Endocrinology, Metabolism and Diabetes of South Africa*, 17:S1–94.

Anjana, R., Pradeepa, R., Deepa, M., Datta, M., Sudha, V., Unnikrishnan, R., Bhanseli, A., Joshi, P., Yajnik, C., Dhandhania, V., Nath, L., Das, A., Rao, P., Madhu, S., Shukla, D., Kaur, T., Priya, M., Nirmal, E., Paryathi, S., Subhashini, S., Subhashini, R., Ali, M. & Mohan, V. (2011). Prevalence of diabetes and prediabetes (impaired fasting glucose and / or impaired glucose tolerance) in urban and rural India : Phase I results of the Indian Council of Medical Research – India Diabetes (ICMR – INDIAB) study. *Diabetologia*, 54:3022–3027.

Asif, M. (2014). The prevention and control of type-2 diabetes by changing lifestyle and dietary pattern. *Journal of Education Health Promotion*, 3:1.

Azevedo, M. & Alla, S. (2008). Diabetes in Sub-Saharan Africa: Kenya, Mali, Mozambique, Nigeria, South Africa and Zambia. *International Journal on Diabetes in Developing*, 28(4):101-8.

Bagonza, J., Rutebemberwa, E. & Bazeyo, W. (2015). Adherence to anti diabetic medication among patients with diabetes in eastern Uganda ; a cross sectional study. *BMC Health Services Research*, 15(168):1–7.

Baliunas, D., Taylor, B., Irving, H., Roerecke, M., Patra, J., Mohapatra, S. & Rehm, J. (2009). Alcohol as a Risk Factor for Type 2. Diabetes Care, 32: 2123–2132.

Barlow, J., Sturt, J. & Hearnshaw, H. (2002). Self-management interventions for people with chronic conditions in primary care: examples from arthritis, asthma and diabetes. *Health Education Journal*, 61(4):365-378.

Berg, C., Rosengren, E., Aires, N., Lappas, G., Torren, K., Thelle, D. & Lissner, L. (2005). Trends in overweight and obesity from 1985 to 2002 in Goteborg, West Sweden. *International Journal of Obesity*, 29:916–924.

Berg, J., Dedd, S. & Dodd, S. (2009). The role of a community pharmacist in diabetes education. *Diabetes Education,* 14(3):148–150.

Blomster, J., Zoungas, S., Chalmers, J., Li, Q., Chow, C., Woodward, M., Mancia, G., Poulter, N., Williams, B., Harrap, S., Neal, B., Patel, A. & Hillis, G.S. (2014). The relationship between alcohol consumption and vascular complications and mortality in Individuals with Type 2 Diabetes. *Diabetes Care*, 37: 1353–1359.

Booysen, B.L. & Schlemmer, A.C. (2015). Reasons for diabetes patients attending Bishop Lavis Community Health Centre being non-adherent to diabetes care *South African Family Practice*, 1–6. Available at: <http://dx.doi.org/10.1080/20786190.2014.977027>.

Bortheiry, A.L., Malerbi, D.A. & Franco, L.J. (1994). The ROC curve in the evaluation of Fasting Capillary Glucose as a screening test for diabetes and IGT. *Diabetes Care*, 17(11): 1269–1272.

Business Tech (2016). The richest and the poorest municipalities in South Africa. Available from: <https://businesstech.co.za/news/wealth/127213/the-richest-and-poorest-municipalities-in-south-africa/>. [Accessed 26^th^ April, 2017.

Cade, W.T. (2008). Diabetes-related microvascular and macrovascular diseases in the physical therapy settings. *Physiotherapy*, 88(11):1322-1335.

Camara, A., Balde, N.M., Sobngwi-Tambekou, J., Kegne, A.P., Diallo, M.M, Tchatchoua, Alain, P.K., Kake, A., Sylvie, N., Balkau, B., Bonnet, F. & Sobngwi, E. (2015). Poor glycemic control in type 2 diabetes in the South of the Sahara : The issue of limited access to an HbA1c test. *Diabetes Research and Clinical Practice*, 108: 187–192.

Carole, D., Suggs, L.S. & Odermatt, P. (2012). Short Message Service (SMS) applications for disease prevention in developing countries. *Journal of Medical Internet Research*, 14(1):e3.

Carratala-Munuera, M.C., Gill-guillen, V.F., Orozio, B.D., Navarro-Perez, J., Caballero-Martinez, F., Alvarez-Guisasola, F., Garcia-Soidon, J., Fluixa-Carrascosa, C., Franch-Nadal, J., Martin-Rioboo, E., Carrillo-Fernandez, L., Artola-Menedez, S., and on behalf of the “Integrated Management of type-2 diabetes” research group. (2015). Barriers associated with poor control in Spanish Diabetes Patients. A Consensus Study. *The International Journal of Clinical Practice*, 67(9):888-894.

Centre for Disease Prevention and Control (2011). National Diabetes Fact Sheet: national estimates and general information on diabetes and pre-diabetes in the United States, Atlanta, GA.

Chawla, A., Chawla, R. & Jaggi, S. (2016). Microvascular and macrovascular complications in diabetes mellitus: Distinct or continuum? *Indian Journal of Endocrinology and Metabolism*, 20(4):546-551.

Cole-Lewis, H., & Kershaw, T. (2010). Text messaging as a tool for behaviour change in disease prevention and management. *Epidemiologic Reviews*, 32(1):56–69.

Cramer, J.A. (2004). A Systematic Review of Adherence With diabetes medication. *Diabetes Care*, 27(5):1224–2004.

Daramola, O.F. (2012). Assessing the validity of random glucose testing for monitoring glycaemic control and predicting HbA1c values in type 2 diabetes at Karl Bremer hospital. Masters Thesis, Stellenbosch University, South Africa.

Dasappa, H., Fathima, F.N., Prabhakar, R. & Sarin, S. (2015). Prevalence of diabetes and pre-diabetes and assessments of their risk factors in urban slums of Bangalore. *Journal of Family Medicine and Primary Care*, 4(6):399–404.

Davies, M.J., Gagliardino, J.J., Gray, L.J., Khunti, K., Mohan, V. & Hughes, R. (2013). Real-world factors affecting adherence to insulin therapy in patients with Type 1 or Type 2 diabetes mellitus : a systematic review. *Diabetic Medicine*, 30: 512–524.

Da-Vita (2016). Diabetes: Definition, Causes and symptoms. Diabetes, 1. Available at: https://www.davita.com/kidney-disease/causes/diabetes/diabetes:-definition,-causes-and-symptoms/e/4991 [Accessed June 2, 2016].

de Tolly, K., Nembaware, V. & Skinner, D. (2011). Encouraging HIV testing through SMS: Can it work, and do content and dosage impact? Durban, South Africa: South African AIDS Conference.

de Vries, S.T., Keers, J.C., Visser, R., Zeeuw, D., De Haaijer-ruskamp, F.M., Voorham, J. & Denig, P. (2014). Medication beliefs, treatment complexity, and non-adherence to different drug classes in patients with type 2 diabetes. *Journal of Psychosomatic Research*, 76:134–138.

Deshpade, A.D., Harres-hages, M. & Schootman, M. (2008). Epidemiology of diabetes and diabetes-related complications. Diabetes Special Issue, *Physical Therapy*, 88(11):1254-1263.

Diabetes Australia (2015). What is diabetes? Diabetes. Available at: https://www.diabetesaustralia.com.au/what-is-diabetes [Accessed June 2, 2016].

Diabetes Uk. Is there a cure for diabetes? Available from: <http://www.diabetes.org.uk/Diabetes-the-basics/is-there-a-cure/>.

Dobson, R., Whittaker, R., Yannan, J., Matthew, S., Maddison, R., Carter, K., Cutfield, R., McNamara, C., Khanolkar, M. & Murphy, R. (2016). Text message-based diabetes self- management support (SMS4BG): study protocol for a randomised controlled trial. *Trials*, 17:179.

Eenfeldt A. (2015). Type 2 diabetes is a fully curable disease. Available from: http://www.dietdoctor.com/type-2-diabetes-is-a-fully-curable disease.

EuroQol Group (2009). EQ-5D-5L, Health Questionnaire. 1-3. Uk.pdf

Ferrer-Roca, O., [Cárdenas](http://journals.sagepub.com/author/C%C3%A1rdenas%2C+A), [A., Diaz-Cardama](http://journals.sagepub.com/author/Diaz-Cardama%2C+A), A. & [Pulido](http://journals.sagepub.com/author/Pulido%2C+P), P. (2004). Mobile phone text messaging in the management of diabetes. *Journal of Telemedicine*, 10:282–285.

Fitzgerald, J.T., Funnell, M.M., Anderson, R.M., Nwankwo, R., Stansfield, R. B. & Piatt, G.A. (2016). Validation of the Revised Brief Diabetes Knowledge Test (DKT2). *The Diabetes Educator*, 42(2):178–187.

Folb N., Timmerman, V., Levitt, N.S., Steyn, K., Bachmann, M.O., Lund, C., Bateman, E.D., Lombard, C., Gaziano, T.A., Zwarenstein, M. & Fairall, L.R. (2015). Multi-morbidity, control and treatment of non- communicable diseases among primary healthcare attenders in the Western Cape, South Africa. *South African Medical Journal*, 105(8):642–647.

Fowler, M.J. (2011). Microvascular and Macrovascular Complications of Diabetes. *Clinical Diabetes*, 29(3):116-122.

Garcia-Perez, L.E., Alvarez, M., Dilla, T., Gil-Guillen, V. & Orozio-Beltran, D. (2013). Adherence to therapies in patients with type-2 diabetes. *Diabetes Therapy,* 4; 175-194.

Guariguata, L., Whiting, D.R., Hambleton, I., Beagley, J., Linnenkamp, U. & Shaw, J.E. (2014). Global estimates of diabetes prevalence for 2013 and projections for 2035. *Diabetes Research and Clinical Practice,* 103(2):137-49.

Hall, C.S. Fortrell, E., Wilkinson, S. & Byass, P. (2014). Assessing the impact of mHealth interventions in low- and middle-income countries what has been shown to work? *Global Health Action*, 7(25606):1–12.

Hall, V., Thomsen, R.W., Henriksen, O. & Lohse, N. (2011). Diabetes in Sub Saharan Africa 1999-2011 : Epidemiology and public health implications: A systematic review. *BMC Public Health*, 11:564.

Hamine, S., Gerth-Guyette W., Faulx D., Green B.B. & Ginsburg, A.S. (2015) Impact of mHealth on chronic disease management on treatment adherence and patient outcomes: A systematic review. *Journal of Medical Internet Research*, 17(2): e52.

Hamsphire, A., Highfield, R.R., Parkin, B.L. & Owen, A.M. (2012). Fractionating Human Intelligence. *Neuron,* 76, 1225–1237.

Health 24 (2016). Medical topics: Diabetes. Available from: http://www.health24.com/Medical/Diabetes/About-diabetes/Diabetes-tsunami-hits-South-Africa-20130210 [Accessed June 3, 2016].

Hex, N., Bartlett, C., Wright, D., Taylor, M. & Varley, D. (2012). Estimating the current and future costs of Type 1 and Type 2 diabetes in the UK, including direct health costs and indirect societal and productivity costs. *Diabetes Medicine*, 855–862.

Hu, F.B. (2011). Globalization of diabetes: The role of diet, lifestyle, and genes. *Diabetes Care*, 34(6): 1249–1257.

Hwang, C.J., Han, P.V., Zabetian, A., Ali, M.K. & Narayan, K.M. (2012). Rural diabetes prevalence quintuples over twenty five years in low-and-middle-income countries: A systematic review and meta-analysis. *Diabetes Research and Clinical Practice*, 96(3): 271–283.

Igbojiaku, O.J., Ogbonnaya C. & Harbor, Ross A. (2013) Compliance with diabetes guidelines at a regional hospital in KwaZulu-Natal, South Africa. *African Journal of Primary Health Care and Family Medicine*. 2013;5(1).

Institute of Medicine. Committee on Health Literacy (2004). Board on Neuroscience and Behavioural Health. Health Literacy: A prescription to End confusion. The National Academic Press: Washington DC.

International Diabetes Federation (2013). IDF Diabetes Atlas 6th ed., Brussels, Belgium: International Diabetes Federation. Available at: <http://www.idf.org/diabetesatlas>.

International Diabetes Federation (2013). IDF Diabetes Atlas 6th ed., Brussels, Belgium: International Diabetes Federation. Available at: <http://www.idf.org/diabetesatlas>.

International Diabetes Federation (2014). IDF Diabetes Atlas - 2014 update. Available at: Dispon?vel em: [www.idf.org/diabetesatlas](http://www.idf.org/diabetesatlas).

International Diabetes Federation (2015a): IDF Diabetes Atlas. In., 7th edn edn. Brussels, Belgium; 1 - 144.

International Diabetes Federation (2015b). Diabetes in South Africa, South Africa. Available from: <http://www.idf.org/membership/afr/south-africa>.

Islam, S.M.S., Lechner, A., Ferrari, U., Froeschl, G., Alam, D.S., Holle, R., Seissler, J.N. & Louis, W. (2014). Mobile phone intervention for increasing adherence to treatment for type 2 diabetes in an urban area of Bangladesh : protocol for a randomized controlled trial. *BMC Health Services Research*, 14:586.

Iwueze J.O. (2007). Managing your Diabetes: Assessment and Management of Patients with Diabetes Mellitus. Owerri: Skillmark Media Ltd: 42.

Jackson, I.L., Adibe, M.O., Okonta, M.J. & Ukwe, C.V. (2015) Medication adherence in type-2 diabetes patients in Nigeria. *Diabetes Technology and Therapeutics*, 14; 431.

Jarab, A.S., Almrayat, R., Alqudah, S., Thehairat, E., Mukattash, T.L., Khdour, M. & Pinto, S. (2014). Predictors of non-adherence to pharmacotherapy in patients with type 2 diabetes. *International Journal of Clinical Pharmacy*, 36: 725–733.

Jemberu, S. (2013). Mobile Health Application in Ethiopia: Existing Initiatives and Practices. Addis Ababa University.

Jha, P., Ramasundarahettige, C., Landsman, V., Rostron, B., Thun, M., Anderson, R.N., Mcafee, T. & Peto, R. (2013). 21st-Century Hazards of Smoking and Benefits of Cessation in the United States. *New England Journal of Medicine*, 368(4): 341–350.

John, W.G. (2012). Use of HbA 1c in the diagnosis of diabetes mellitus in the UK. The implementation of World Health Organization guidance 2011. *Diabetic Medicine*, 29(11): 1350–1357.

Joint united Nations Programme on HIV/AIDs (2005). Resource needs for an expanded response to AIDS in low- and middle-income countries, Geneva, Switzerland.

Kadima, F.N. & Tumbo, J.M. (2013) Screening of long-term complications and glycaemic control of patients with diabetes attending Rustenburg Provincial Hospital in North West Province, South Africa. *African Journal of Primary Health Care and Family Medicine*, 5(1).

Kagee, E. (2004). Treatment adherence in South African primary health care. *South African Family Practice*, 10(26-30).

Kanavos, P., Van der Aardweg, S. & Schurer, W. (2012). Diabetes expenditure, burden of disease and management in 5 EU countries, London.

Kassahun, T., Eshetie, T. & Gesesew, H. (2016). Factors associated with glycemic control among adult patients with type 2 diabetes mellitus : a cross ‑ sectional survey in Ethiopia. *BMC Research Notes*, 9:78.

Khan, N.A., Venkatachalam, V.V., Al, Khaled M., Sirajudeen, A., Dhanapal, C.K., Ansari, A. & Mohammad, S. (2015). Overview of Glycaemic control, knowledge and attitude among type-2 diabetes male patients. *Journal of Applied Pharmaceutical Science*, 7(1):75–82.

Khattab, A., Javaid, A., Iraqi, G., Alzaabi, A., Ben, A., Koniski, M., Shahrour, N., Taright, S., Idrees, M., Polatli, M., Rashid, N. & El Hasnaoui, A. (2012). Smoking habits in the Middle East and North Africa : Results of the BREATHE study. *Respiratory Medicine*, 106(S2): S16–S24.

Kibirige, D., Atuhe, D., Sebunya, R. & Mwebaze, R. (2014). Suboptimal glycaemic and blood pressure control and screening for diabetic complications in adult ambulatory diabetic patients in Uganda : a retrospective study from a developing country. *Journal of Diabetes & Metabolic Disorders*, 13(40):1–7.

Kirkman, M.S., Briscoe, V.J., Clark, N., Florez, H., Haus, L.B. & Halter, J.B. (2012). Diabetes in older adults. *Diabetes Care*, 35:2650-2664.

Krishna, S. & Boren, S.A. (2008). Diabetes Self-Management Care via Cell Phone: A Systematic Review. *Journal of Diabetes Science and Technology*, 2(3):509–517.

Kruijshoop, M., Feskens, E.J.M., Blaak, E.E. & de Bruin, T.W.A. (2004). Validation of capillary glucose measurements to detect glucose intolerance or type 2 diabetes mellitus in the general population. *International Journal of Clinical Chemistry*, 341(1-2): 33–40.

Leon, N., Surender, R., Bobrow, K., Muller, J. & Farmer, A. (2015). Improving treatment adherence for blood pressure lowering via mobile phone SMS-messages in South Africa : a qualitative evaluation of the SMS-text Adherence support. *BMC Family Practice*, 16(80):1–10.

Lester, R.T., Gelmon, L. & Plummer, F.A. (2006). Cell phones: tightening the communication gap in resource-limited antiretroviral programmes? *AIDS*, 20(17):2242–2244.

Leventhal, H. & Cameron, L. (1987): Behavioural theories and the problem of compliance. *Patient Education and Counselling*, 10(2):117–138.

Lorig, K., Ritter, P.L. & Plant, K. (2005). A disease-specific self-help program compared with generalized chronic disease self-help program for arthritis patients. *Journal of Arthritis and Rheumatism*, 53(6):950-957.

Lun, S., Yeung, A., Jiang, C., Cheng, K., Cowling, B., Liu, B., Zhang, W., Lam, T., Leung, G. & Schooling, C. (2013). Moderate alcohol use and cardiovascular disease from mendelian randomization. *PloS ONE*, 8(7):e68054.

Lunny, C., Taylor, D., Memetovic, J., Wärje, O., Lester, R., Wong, T., Ho, K., Gilbert, M. & Ogilvie, G. (2014). Short message service (SMS) interventions for the prevention and treatment of sexually transmitted infections : a systematic review protocol. *Systematic Reviews*, 3(7):1–8.

Mandewo, W., Dodge, E.E., Chideme-Munodawafa, A. & Mandewo, G. (2014). Non-adherence to treatment among diabetic patients attending outpatients clinic at Mutare Provincial Hospital, Manicaland Province, Zimbabwe. *International Journal of Scientific and Technology Research*, 3(9):66–86.

Mann, D.M., Ponieman, D., Leventhal, H. & Halm, E.A. (2009). Predictors of adherence to diabetes medications : the role of disease and medication beliefs. *Journal of Behavioural Medicine*, 32:278–284.

Manyema, M., Veerman, L., Chola, L., Tugendhaft, A., Labadarios, D. & Karen H. (2015). Decreasing the burden of type 2 diabetes in South Africa : The impact of taxing sugar- sweetened beverages. *Plos One*, 10(11):e0143050.

Marwaha, A. (2010). Health advice smartphone app launches in South Africa. Technology. Available at: <http://www.bbc.co.uk/news/10407081>.

Mash, B., [Ponieman, D](https://www.ncbi.nlm.nih.gov/pubmed/?term=Ponieman%20D%5BAuthor%5D&cauthor=true&cauthor_uid=19184390)., [Leventhal, H](https://www.ncbi.nlm.nih.gov/pubmed/?term=Leventhal%20H%5BAuthor%5D&cauthor=true&cauthor_uid=19184390). & [Halm, E.A](https://www.ncbi.nlm.nih.gov/pubmed/?term=Halm%20EA%5BAuthor%5D&cauthor=true&cauthor_uid=19184390). (2012). Effectiveness of a group diabetes education programme in underserved communities in South Africa : pragmatic cluster randomized control trial. *BMC Family Practice*, 13(126):1–7.

Mason, K. (2011). Providing diabetes health coverage: state laws & programs, United States of America. Available at: <http://www.ncsl.org/research/health/diabetes-health-coverage-state-laws-and-programs.aspx>.

Mbanya, J.C.N., Motala, A.A., Sobngwi, E., Assah, F.K. & Enoru, S.T. (2010). Diabetes in sub-Saharan Africa. *The Lancet*, 375: 2254–2266.

Mbuagbaw, L., Lehana, T., Ongolo-Zogo, P., Lester, R.T., Mills, E.J., Smieja, M., Dolovich, L. & Kouanfack, C. (2012). The Cameroon Mobile Phone SMS (CAMPS) Trial : a randomized trial of text messaging versus usual care for adherence to antiretroviral therapy. *PLoS ONE*, 7(12):e46909.

Motala, A. & Ramaya, K. (2010). Diabetes : the hidden pandemic and its impact on sub-Saharan Africa, Novo Nordisk, Johannesburg, South Africa.

National Health System (2016). Diabetes type 2: Diagnosis. Diabetes mellitus. Available at: http://www.nhs.uk/Conditions/Diabetes-type2/Pages/Diagnosis.aspx [Accessed June 6, 2016].

National Institute of Diabetes and Digestive and Kidney Diseases (2014). Physical activity and diabetes mellitus, Bethesda. Available at: <https://www.niddk.nih.gov/health-information/diabetes>.

National Institute of Diabetes and Digestive and Kidney Diseases (2016). Diagnosis of diabetes and pre-diabetes. Health Information. Available at: http://www.niddk.nih.gov/health-information/health-topics/Diabetes/diagnosis-diabetes-prediabetes/Pages/index.aspx [Accessed June 3, 2016].

Nemeh, A.A., Khader, Y. & Aysha, A. (2011). Glycaemic control and its determinants among patients with type 2 diabetes mellitus attending a teaching hospital. *Journal of Diabetes and Metabolism*, 2(4):1000129.

Nolan, C.J., Damm, P. & Prentki, M. (2011). Type 2 diabetes across generations: from pathophysiology to prevention and management. *Lancet*, 378(9786): 169–181.

Norman, R., Bradshaw, D., Schneider, M., Pieterse, D. & Groenewald, P. (2006). Revised burden of disease estimate for the comparative risk factor assessment, South Africa 2000. Methodological Note. Cape Town: South African Medical Research Council.

Owolabi, E.O., Goon, D.T., Adeniyi, O.V. & Seekoe, E. (2016). Correlates of pre-diabetes and type 2 diabetes in Buffalo City Municipality, South Africa. *African Journal for Physical Activity and Health Sciences*, 22(4:1):1019-1035.

Pal, K., Eastwood, S.V., Michie, S., Farmer, A.J., Barnard, M.L., Peacock, R., Wood, B., Inniss, J.D., Murray, E., Barnard, M.I. & Murray, E. (2013). Computer-based diabetes self-management interventions for adults with type 2 diabetes mellitus (Review). *Cochrane Database of Systematic Reviews*, CD00876(3):1–150.

Park, A. (2011). Why smoking is especially bad if you have diabetes. Tobacco. Available at: http://healthland.time.com/2011/03/27/why-smoking-is-a-bad-idea-for-diabetics/ [Accessed June 22, 2016].

Pinto, G. & Beltrán-sánchez, H. (2015). Prospective study of the link between overweight / obesity and diabetes incidence among Mexican older adults : 2001-2012. *Salud Publica de Mexico*, 57(1): S15–S21.

Rwegerera, G.M. (2014). Adherence to anti-diabetic drugs among patients with Type 2 diabetes mellitus at Muhimbili National Hospital, Dar es Salaam, Tanzania- A cross-sectional study. *Pan African Medical Journal*, 8688:1–9.

Ryan, P. (1988).*Interventions to facilitate behaviour change*. Marquette University College of Nursing, Milwankee, WI:

Sankar, U.V., Kasia, L., Mini, G.K., Sarma, P.S. & Thankappan, K.R. (2015). The adherence to medications in diabetic patients in rural Kerala, India. *Asia Pacific Journal of Public Health*, 27(2):941-957.

Schrieks, I., Heil, A., Hendriks, H., Mukamal, K. & Beulens, J. (2015). The effect of alcohol consumption on insulin sensitivity and glycaemic status : A systematic review and meta-analysis of intervention studies. *Diabetes Care*, 38(4): 723–732.

Schwellnus, M.P., Patel D.N., Nossel, C., Dreyer, M., Whitesman, S. & Derman, E.W. (2009). Healthy lifestyle interventions in general practice Part 4 : Lifestyle and diabetes mellitus. *South African Family Practice*, 51(1):19–25.

Seuring, T., Archangelidi, O. & Suhrcke, M. (2015). The economic costs of type 2 diabetes : A global systematic review. *PharmacoEconomics*, 33: 811–831.

Shen, J., [Rubinstein, A](https://www.ncbi.nlm.nih.gov/pubmed/?term=Rubinstein%20A%5BAuthor%5D&cauthor=true&cauthor_uid=27102023)., [Irazola, V](https://www.ncbi.nlm.nih.gov/pubmed/?term=Irazola%20V%5BAuthor%5D&cauthor=true&cauthor_uid=27102023)., [Gutierrez, L](https://www.ncbi.nlm.nih.gov/pubmed/?term=Gutierrez%20L%5BAuthor%5D&cauthor=true&cauthor_uid=27102023)., [Miranda, J.J](https://www.ncbi.nlm.nih.gov/pubmed/?term=Miranda%20JJ%5BAuthor%5D&cauthor=true&cauthor_uid=27102023)., [Bernabé-Ortiz, A](https://www.ncbi.nlm.nih.gov/pubmed/?term=Bernab%C3%A9-Ortiz%20A%5BAuthor%5D&cauthor=true&cauthor_uid=27102023)., [Lazo-Porras, M](https://www.ncbi.nlm.nih.gov/pubmed/?term=Lazo-Porras%20M%5BAuthor%5D&cauthor=true&cauthor_uid=27102023)., [Levitt. N](https://www.ncbi.nlm.nih.gov/pubmed/?term=Levitt%20N%5BAuthor%5D&cauthor=true&cauthor_uid=27102023)., [Steyn, K](https://www.ncbi.nlm.nih.gov/pubmed/?term=Steyn%20K%5BAuthor%5D&cauthor=true&cauthor_uid=27102023)., [Bobrow, K](https://www.ncbi.nlm.nih.gov/pubmed/?term=Bobrow%20K%5BAuthor%5D&cauthor=true&cauthor_uid=27102023)., [Ali, M.K](https://www.ncbi.nlm.nih.gov/pubmed/?term=Ali%20MK%5BAuthor%5D&cauthor=true&cauthor_uid=27102023)., [Prabhakaran, D](https://www.ncbi.nlm.nih.gov/pubmed/?term=Prabhakaran%20D%5BAuthor%5D&cauthor=true&cauthor_uid=27102023). & Tandon, N. (2016). A multi-ethnic study of pre-diabetes and diabetes in LMIC. *Global Heart*, 11(1):61–70.

Shilubane, N. (2010). Factors contributing to poor glycaemic control in diabetic patients at Mopani District. *Curationis*, 33(3):43–47.

Shrivastava, S.R., Shrivastava P.S. & Ramasamy, J. (2013). Role of self-care in management of diabetes mellitus. *Journal of Diabetes and Metabolic Syndrome*, 5; 12(1):14.

Sinha, S.R. & Barry, M. (2011). Health technologies and innovation in the global health arena. *National England Journal of Medicine*, 365(9):779–82.

Stratton, I.M., Adler, A., Neil, H.A.W., Matthew, D.R., Manley, S.E., Cull, C.A., Haddon, D., Turner, R.C. & Holman, R.R.(2000). Association of glycaemia with macrovascular and prospective observational study. *BioMedical Journal*, 321:405–412.

Teoh H, Braga MF, Casanova A, et al. (2010). Patient age, ethnicity, medical history, and risk factor profile, but not drug insurance coverage, predict successful attainment of glycemic targets: Time 2 Do More Quality Enhancement Research Initiative (T2DM QUERI). Diabetes Care, 33:2558–60.

The ACCORD Study Group & ACCORD Eye Study Group (2010). Effects of medical therapies on retinopathy progression in type 2 diabetes. *New England Journal of Medicine*, 363(3): 33–244.

The Diabetes Control and Complications Trial Research Group (1993). The effect of intensive treatment of diabetes on the development and progression of long-term complications in insulin dependent diabetes mellitus. *The New England Journal of Medicine*, 329(14):977–986.

Uchenna, V., Ijeoma, O., Peace, N. & Ngozi, K. (2009). Knowledge of diabetes management and control by diabetic patients at Federal Medical Center Umuahia Abia State, Nigeria. *International Journal of Medicine and Medical Sciences*, 1(9):353–358.

Webb, E.M., Rheeder, P. & Zyl, D.G. Van (2014). Diabetes care and complications in primary care in the Tshwane district of South Africa. *Primary Care Diabetes*, 9(2):147–154.

Whiting, D., Guarigata, L., Weil, C. & Shaw, J. (2011). IDF Diabetes Atlas: Global estimates of the prevalence of diabetes for 2011 and 2030. *Diabetes Research and Clinical Practice*, 94:311–321.

Willi, C., Bondenmann, P. & Ghali, W.A. (2007). Active smoking and the risk of type 2 diabetes: a systematic review and meta-analysis. *Journal of the American Medical Association*, 298(22): 2654–2664.

Woldu, M.A. Wami, C.D., Lenjisa, J.L., Tegegne, G.T., Tesafye, G. & Dinsa, H. (2014). Factors associated with poor glycaemic control among patients with type 2 diabetes mellitus in Ambo Hospital, Ambo, Ethiopia. *Endocrinology and Metabolic Syndrome*, 3(4):1–6.

World Health Organization (2006). Definition and diagnosis of diabetes mellitus and intermediate hyperglycemia, Geneva.

World Health Organization (2010). Global recommendations on physical activity for health, Geneva. Available at: (http://whqlibdoc.who.int/publications/2010/9789241599979_eng.pdf.

World Health Organization (2011). Use of glycated haemoglobin (HbA1c) in the diagnosis of diabetes mellitus: abbreviated report of a WHO consultation, Available at: <http://www.who.int/diabetes/publications/report-hba1c_2011.pdf>.

World Health Organization (2014a). World health statistics 2014. Available at: (http://apps.who.int/iris/ [Accessed May 23, 2015].

World Health Organization (2014b). The Top Ten leading causes of deaths in the world, 2000 and 2012. WHO factsheets. Available at: http://www.who.int/mediacentre/factsheets/fs310/en/ [Accessed January 12, 2016].

World Health Organization (2016a). Global report on diabetes, Geneva, Switzerland.

World Health Organization (2016b). World Health Day 2016: Beat Diabetes. Diabetes, pp.1–2. Available at: http://www.who.int/campaigns/world-health-day/2016/en/ [Accessed June 7, 2016].

World Health Organization (2016b). World Health Day 2016: Beat Diabetes. Diabetes, pp.1–2. Available at: http://www.who.int/campaigns/world-health-day/2016/en/ [Accessed June 7, 2016].

World Health Organization (2016c). Diabetes mellitus. Factsheet. Available at: http://www.who.int/mediacentre/factsheets/fs138/en/ [Accessed June 3, 2016].

World Heart Federation (2016). Obesity. Cardiovascular risk. Available at: http://www.world-heart-federation.org/cardiovascular-health/cardiovascular-disease-risk-factors/obesity/ [Accessed June 15, 2016].

Yang, X., Hsu-Hage, B., Yu, L. & Simmons, D. (2002). Selective Screening for Gestational Diabetes in Chinese Women. *Diabetes Care*, 24(4): 798–802.

Zhao, X., Zhao, W., Zhang, H., Li, J., Shu, Y., Li, S., Cai, L., Zhou, J., Li, Y. & Hu, R. (2013). Fasting capillary blood glucose: An appropriate measurement in screening for diabetes and pre-diabetes in low-resource rural settings. *Journal of Endocrinology Investigation*, 36(1): 33–37.
