# Supplementary material for: Efficacy, acceptability and feasibility of daily text-messaging in promoting glycaemic control and other clinical outcomes in a low-resource setting of South Africa: A randomised controlled trial
Source: PLoS One. 2019 Nov 27;14(11):e0224791. doi: 10.1371/journal.pone.0224791 (PMC6881007; doi:10.1371/journal.pone.0224791)
Supplement: S4 File — (DOCX) [file pone.0224791.s004.docx]

**SECTION A : Demographic Information**

| **Demographic Information** | | | |
| --- | --- | --- | --- |
| **Question** | | **Response** | **Code** |
| **1** | Sex | Male 1  Female 2 | C1 |
| **2** | What is your date of birth? | └─┴─┘ └─┴─┘ └─┴─┴─┴─┘  dd mm year | C2 |
| **3** | What is the **highest level of education** you have completed? | No formal schooling 1 | C5 |
|  |  | Grade 1-7 2 |  |
|  |  | Grade 8-12 3 |  |
|  |  | Tertiary 4 |  |
|  |  | Post-graduate degree 5 |  |
| **4** | What is your racial group? | Black 1  Coloured 2  White 3 | C6 |
| **5** | What is your **marital status**? | Never married 1  Married 2  Divorced 3  Widowed 4  Cohabiting 5 | C7 |
| **6.** | Which of the following best describes your **main work** status over the past 12 months? | Government employee 1  Non-government employee 2  Self-employed 3  Student 4  Retired 5  Unemployed 6 | C8 |
| **7** | When did you commence diabetes treatment? | ____________________ | C9 |
| **8** | Type of Diabetes? | Type 1 1  Type 2 2 |  |
| **9.** | What type of treatment are you receiving for your diabetes? | Oral pills 1  Insulin 2 |  |
| **10** | If you are on oral pills, mention the name of the drugs you are currently using | __________________________ |  |

**SECTION B Behavioural Measurements**

| **Tobacco Use** | | | | |  |
| --- | --- | --- | --- | --- | --- |
| **1** | Have you ever smoked any tobacco product? | Yes 1  No 2 *If No, go to T6* | | T1 |  |
| **2** | Do you currently smoke any **tobacco products**, such as cigarettes, cigars or pipes? | Yes 1  No 2 *If No, go to T6* | | T1 |  |
| **3** | Do you currently smoke tobacco products **daily**? | Yes 1  No 2 *If No, go to T6* | | T2 |  |
| **4** | On average, **how many** of the following do you smoke each day? | Manufactured cigarettes **└─┴─┘** | | T5a |  |
|  |  | Hand-rolled cigarettes **└─┴─┘** | | T5b |  |
|  |  | Pipes full of tobacco **└─┴─┘** | | T5c |  |
|  |  | Snuff └─┴─ | | T5d |  |
| **5** | During the past 7 days, on how many days did someone **in your home** smoke when you were present? | Number of days [ ] | | T13 |  |
| **6** | During the past 7 days, on how many days did someone smoke in closed areas **in your workplace** (in the building, in a work area or a specific office) when you were present? | Number of days [ ] | | T14 |  |
|  |  |  |  |  |  |
| **Alcohol Consumption** | | | | |  |
| **7** | Have you **ever** consumed an alcoholic drink | | Yes 1  No 2 *If No, go to D1* | A1a |  |
| **8** | Have you consumed an alcoholic drink within the **past 30days**? | | Yes 1  No 2 *If No, go to D1* | A1b |  |
| **9** | During the past 30days, **how frequently** have you had at least one alcoholic drink? | | Daily 1  5-6 days per week 2  1-4 days per week 3  1-3 days per month 4  Less than once a month 5 | A2 |  |
| **10** | During the past 30 days, how many times did you have  for **men**: **five or more**  for **women**: **four or more**  standard alcoholic drinks in a single drinking occasion? | | Number of times └─┴┘ | A7 |  |

| **Dietary Practices**  **: Diet** | | | |
| --- | --- | --- | --- |
|  | |  |  |
| **11** | Have you ever been taught about the recommended diets for diabetes? know about the diabetes diet plate? (Show an example) | Yes 1  No 2 |  |
| **12** | If yes, do you comply with the recommendations? | Yes 1  No 2 |  |
| 13 | Looking at this diabetes plate, do you feel you eat more than the recommended amount? | Yes 1  No 2 |  |
| 14 | If you do not comply, why? |  |  |
| **CORE: Physical Activity** | | | |
| **Question** | | Response | Code |
| **15** | Do you participate in moderate physical activities? | Yes 1  No 2 | P1 |
| **16** | Do you think you are active enough? | Yes 1  No 2 | P2 |
| **17** | Do you comply with all the recommended physical activity pattern? | Yes 1  No 2 | P3 |
| **18** | If not, why? |  | P4 |

| **Sedentary behaviour** | | | |
| --- | --- | --- | --- |
| The following question is about sitting or reclining at work, at home, getting to and from places, or with friends including time spent sitting at a desk, sitting with friends, traveling in car, bus, train, reading, playing cards or watching television, but do not include time spent sleeping. | | | |
| **20** | How much time do you usually spend sitting or reclining on a typical day? | └─┴─┘:└─┴┘  Hours : minutes | P16(a-b) |

**SECTION C: HISTORY TAKING**

| **History of Raised Blood Pressure** | | | |  |
| --- | --- | --- | --- | --- |
| **1** | Have you ever been told by a doctor or other health worker that you have raised blood pressure or hypertension? | Yes 1  No 2 *If No, go to H6* | H2a |  |
| **2** | Are you currently receiving any of the following treatments/advice for high blood pressure prescribed by a doctor or other health worker? | | |  |
|  | Drugs (medication) that you have taken in the past two weeks | Yes 1  No 2 | H3a |  |
| **History of Diabetes** | | | | |
| **3** | Are you currently receiving any of the following treatments/advice for diabetes prescribed by a doctor or other health worker? | | | |
|  | Insulin | Yes 1  No 2 | H8a | |
|  | Drugs (medication) that you have taken in the past two weeks | Yes 1  No 2 | H8b | |
| **4.** | What are the drugs you are currently using for your diabetes? |  |  | |
| **5.** | Does any member of your family have diabetes? | Yes 1  No 2 |  | |
| **6.** | If yes, who? |  |  | |

**SECTION L: PERCEPTION ABOUT THE USE OF MHEALTH**

**1.** Do you feel the use of mobile phone technology can assist you in any way regarding your diabetes treatment?

a. Yes [ ] b. No [ ]

**2.** Do you feel it is a waste of time?

a. Yes [ ] b. No [ ]

**3.** Are you happy with receiving SMSs daily regarding your diabetes care?

a. Yes [ ] b. No [ ]

**4.** Briefly tell us how you feel about it.

................................................................................................................................................................................................................................................................................................................................................................................................................................................................................................................................................................................................................................................................................................................................................................................................................

**ANTHROPOMETRIC, PHYSICAL AND BIO-CHEMICAL MEASUREMNTS**

|  | **1^ST^ ATTEMPT** | **2^ND^ ATTEMPT** | **3^rd^ ATTEMPT** |
| --- | --- | --- | --- |
| **Weight(Cm)** |  |  |  |
| **Height(m)** |  |  |  |
| **Waist circumference(Cm)** |  |  |  |
| **Hip circumference(Cm)** |  |  |  |
| **Blood pressure (mmHg)** |  |  |  |
| **HbA1c (%)** |  | | |
| **Lipid** |  | | |

**ACCEPTABILITY OF SMS INTERVENTION**

| **SN** | **QUESTION** |  |
| --- | --- | --- |
| 1 | DID YOU RECEIVE THE DAILY SMS? | Yes  No |
| 2 | IF YES, DO YOU THINK IT WAS HELPFUL? |  |
| 3 | IN WHAT WAY WAS IT HELPFUL? EXPLAIN |  |
|  | | |
| 4 | DID IT STRESS YOU IN ANY WAY? | Yes  No |
| 5 | IF YES, HOW DID IT STRESS YOU? |  |
| 6 | WERE YOU COMFORTABLE WITH THE TIMING OF THE SMS? | Yes  No |
| 7 | IF NOT, WHY? |  |
| 9 | IF WE DECIDE TO CONTINUE, WOULD YOU LIKE TO CONTINUE? | Yes  No |
| 10 | WHAT WOULD YOU LIKE US TO IMPROVE UPON REGARDING THE SMS? |  |
